# A conserved folding nucleus sculpts the free energy landscape of bacterial and archaeal orthologs from a divergent TIM barrel family

**DOI:** 10.1073/pnas.2019571118

**Published:** 2021-04-19

**Authors:** Rohit Jain, Khaja Muneeruddin, Jeremy Anderson, Michael J. Harms, Scott A. Shaffer, C. Robert Matthews

**Affiliations:** ^a^Department of Biochemistry and Molecular Pharmacology, University of Massachusetts Medical School, Worcester, MA 01605;; ^b^The Mass Spectrometry Facility, University of Massachusetts Medical School, Shrewsbury, MA 01545;; ^c^Department of Chemistry and Biochemistry, University of Oregon, Eugene, OR 97403

**Keywords:** protein folding, protein evolution, TIM barrel orthologs, hydrogen deuterium exchange, mass spectrometry

## Abstract

The amino acid sequences of proteins have evolved over billions of years, preserving their structures and functions while responding to evolutionary forces. Are there conserved sequence and structural elements that preserve the protein folding mechanisms? The functionally diverse and ancient (βα)_1–8_ TIM barrel motif may answer this question. We mapped the complex six-state folding free energy surface of a ∼3.6 billion y old, bacterial indole-3-glycerol phosphate synthase (IGPS) TIM barrel enzyme by equilibrium and kinetic hydrogen–deuterium exchange mass spectrometry (HDX-MS). HDX-MS on the intact protein reported exchange in the native basin and the presence of two thermodynamically distinct on- and off-pathway intermediates in slow but dynamic equilibrium with each other. Proteolysis revealed protection in a small (α1β2) and a large cluster (β5α5β6α6β7) and that these clusters form cores of stability in I_a_ and I_bp_. The strongest protection in both states resides in β4α4 with the highest density of branched aliphatic side chain contacts in the folded structure. Similar correlations were observed previously for an evolutionarily distinct archaeal IGPS, emphasizing a key role for hydrophobicity in stabilizing common high-energy folding intermediates. A bioinformatics analysis of IGPS sequences from the three superkingdoms revealed an exceedingly high hydrophobicity and surprising α-helix propensity for β4, preceded by a highly conserved βα-hairpin clamp that links β3 and β4. The conservation of the folding mechanisms for archaeal and bacterial IGPS proteins reflects the conservation of key elements of sequence and structure that first appeared in the last universal common ancestor of these ancient proteins.

## Significance

Orthologous proteins from the three superkingdoms have conserved their structures and functions over evolutionary time. We ask whether their folding mechanisms and the structures of their partially folded states are similarly conserved, using bacterial and archaeal representatives of the IGPS TIM barrel enzyme. Comparison of circular dichroism and fluorescence spectroscopic studies reveal a highly conserved mechanism, and hydrogen–deuterium exchange mass spectrometry analyses highlight similar cores of stability in regions dominated by clusters of branched aliphatic side chains. A bioinformatics analysis of hundreds of IGPS sequences from each superkingdom shows a very highly conserved sequence, V/ILLI, that nucleates the formation of a misfolded, microsecond intermediate and has existed since the last universal common ancestor of the IGPS family of proteins.

Proteins are indispensable workhorses of cellular machinery whose functional diversity is defined by their final folded conformations. The folding pathway of a protein is determined by its energy landscape, whose map is encoded in the amino acid sequence. Partially folded states on the landscape often contain elements of the native topology and connect the nascent unfolded polypeptide chain to the functional folded conformation (1, 2). Proteins and their folding pathways have evolved over billions of years, responding to evolutionary forces such as mutation and natural selection (3–5). Orthologs, proteins that have diverged from a common ancestor but share a common structure and function, provide vehicles for exploring the impact of evolution on folding pathways and the intermediates that guide the folding to the native conformation.

The functionally diverse (βα)_1–8_ TIM barrel motif is an ideal candidate to decipher evolutionary constraints on protein folding pathways. The motif supports a wide variety of essential enzymatic transformations in all three superkingdoms of life (6–8) and is one of the 10 ancestral protein folds that were instrumental in the transition from RNA–protein world to the last universal common ancestor of life (LUCA) to the present complex DNA–RNA–protein world (9, 10). The βα-repeat architecture produces a cylindrical β-barrel core and an amphipathic α-helical shell whose loops between the β-strands and subsequent α-helices form the canonical active site of this very large family of enzymes. Although the pairwise sequence conservation across the family of TIM barrels is typically ∼30%, their folding mechanisms are complex and highly conserved (11). Folding intermediates, both on the productive folding pathway and as misfolded, kinetic traps have been observed for candidate TIM barrels from several bacterial and archaeal organisms (11–16). The divergence of these two superkingdoms, which occurred ∼4 billion y ago, right after life arose, speaks to the robustness of the TIM barrel folding mechanism across the span of evolutionary time.

We have previously examined the relationships between sequence, structure, and fitness in a yeast-based competition assay for three thermophilic indole-3-glycerolphosphate synthase (IGPS) orthologs from the TIM barrel family (17). Significant correlations between the archaeal *Sulfolobus solfataricus* (SsIGPS) and the bacterial *Thermotoga maritima* (TmIGPS) and *Thermus thermophilus* (TtIGPS) proteins revealed that both sequence and structure are critical in defining their fitness landscapes. This observation and the conservation of TIM barrel folding mechanisms motivated the hypothesis that the sequences of TIM barrel orthologs from archaeal and bacterial organisms also conserve the structures of their folding intermediates. If valid, we would obtain detailed insights into the constraints that TIM barrel structure and function impose on the enormous sequence space available in ∼4 billion y of evolution (18, 19). We have previously mapped the structures of the on- and off-pathway intermediates for SsIGPS by hydrogen–deuterium exchange mass spectrometry (HDX-MS) (15, 16), providing an archaeal reference for the present study of a bacterial ortholog (*SI Appendix*, Fig. S1).

Comparison of the structures of the folding intermediates and folding mechanisms for *S. solfataricus* and *T. maritima* IGPS confirmed our hypothesis. A bioinformatics analysis of thousands of nonredundant IGPS sequences from the bacterial, archaeal, and eukaryota superkingdoms revealed the conservation of three adjacent structural elements that form a nucleus responsible for defining the folding free energy surface of the IGPS family of TIM barrel proteins. We conclude that the folding mechanism of the IGPS TIM barrel, including the structures of key partially folded states, arose in the LUCA and has persisted for over ∼4 billion y.

## Results

### The Folding Mechanism Is Conserved in Bacterial and Archaeal IGPS TIM Barrels.

#### Equilibrium experiments.

**Fig. 1.**
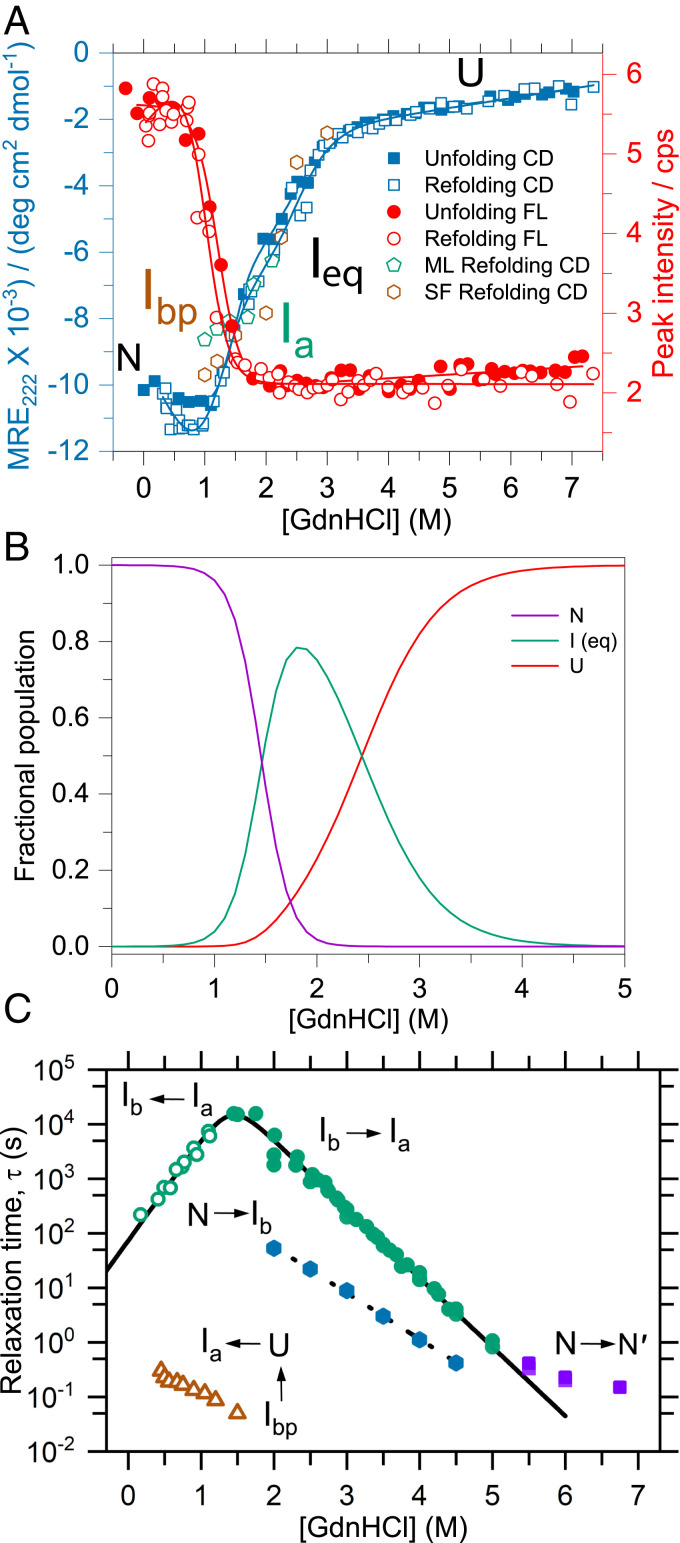
Thermodynamic and kinetic folding properties of TmIGPS. (*A*) Titration curves are shown after 9 d equilibration in GdnHCl (*SI Appendix*, Fig. S2) and were fitted to a three-state CD model (blue) and a two-state FL model (red). A large burst phase was seen after 10 s for the on-pathway intermediate, I_a_, in manual (green) and after 10 ms for the off-pathway intermediate, I_bp_, in stopped-flow (brown) refolding CD experiments at different GdnHCl concentrations. (*B*) The fractional population plot is based on thermodynamic parameters extracted from a fit of the CD data to an apparent three-state model (*SI Appendix*, Table S1). (*C*) A semilog plot of the relaxation times, τ, acquired from unfolding (filled symbols) and refolding (open symbols) experiments is plotted against final GdnHCl concentration. The solid line for slow unfolding and refolding phases is a fit to a two-state chevron model (53). The assignments of the phases to specific steps in the mechanism are indicated.

We monitored the equilibrium and kinetic folding properties of TmIGPS in the denaturant guanidine hydrochloride (GdnHCl) with circular dichroism (CD) and tryptophan fluorescence (FL) spectroscopy to monitor the formation and disruption of secondary and tertiary structure (Fig. 1). Surprisingly, the titrations of TmIGPS with GdnHCl took 9 d to reach equilibrium at 25 °C and pH 7.2 (*SI Appendix*, Fig. S2). The CD results at 222 nm revealed a shoulder at ∼2 M GdnHCl and follow an apparent three-state mechanism, N ↔ I_eq_ ↔ U, similar to other TIM barrel proteins (13, 14). By contrast, FL spectroscopy only detected the N ↔ I_eq_ transition (Fig. 1*A*). The I_eq_ state retains ∼50% of the far-ultraviolet (UV) CD signal, but the α6 (W194) and α8 (W250) helices containing the two tryptophans appear to be unfolded. The population of I_eq_ is highest at 1.8 M GdnHCl, ∼80% of the population, and transition to the unfolded state is complete by ∼5 M GdnHCl (Fig. 1*B*). To enhance the precision of the thermodynamic parameters from each technique, we independently fitted the CD ellipticities between 200 and 260 nm to a three-state model and the FL emission data between 305 and 450 nm to a two-state model. The CD data yielded a free energy for the N ↔ I_eq_ transition of 5.4 ± 0.1 kcal ⋅ mol^−1^, and 4.1 ± 0.2 kcal ⋅ mol^−1^ for the I_eq_ ↔ U transition (*SI Appendix*, Table S1). FL spectroscopy yielded an apparent free energy for the N ↔ I_eq_ transition of 4.9 ± 0.6 kcal ⋅ mol^−1^, in excellent agreement with the CD measurement.

#### Kinetic experiments.

To obtain a complete picture of the folding reaction, we complemented the equilibrium results with an analysis of the kinetic unfolding and refolding properties of TmIGPS over a time range of milliseconds to hours. The kinetic traces were fitted to one or two exponential functions, and the log_10_ of the observed relaxation times were plotted as a function of the final GdnHCl concentration (Fig. 1*C*). TmIGPS folds in the submillisecond time frame to a kinetic intermediate, I_bp_, with substantial secondary structure (Fig. 1*A*). The denaturant dependence of the I_bp_ ellipticity is coincident with the I_eq_ ↔ U transition, implying an apparent stability comparable to I_eq_ (Fig. 1*A*). Unfortunately, aggregation below 1 M GdnHCl precluded a quantitative analysis of its stability. Stopped-flow FL (SF-FL) revealed a 100’s of millisecond reaction whose acceleration at increasing denaturant concentration indicates an unfolding-like reaction for I_bp_ (Fig. 1*C*). The final, rate-limiting step in refolding accelerates exponentially with decreasing GdnHCl to reach an extrapolated relaxation time of 74 s in the absence of denaturant. The unfolding SF-FL kinetic traces were fitted with two exponential functions and describe a pair of denaturant-dependent phases between 2 and 4.5 M GdnHCl. The major (∼95%) slow and minor (∼5%) fast unfolding phases merged into a single phase whose relaxation time rolls over at ≥5 M GdnHCl. Although the major FL unfolding phase is also detected by CD, the minor unfolding phase is not.

**Scheme 1.**
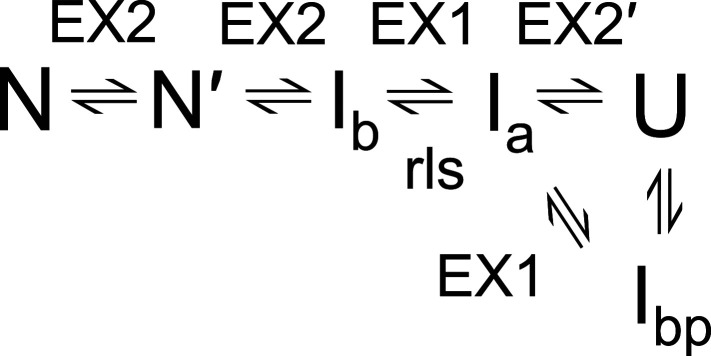


We interpret the kinetic results to support a six-state folding mechanism for TmIGPS (Scheme 1), very similar to those for other IGPS TIM barrels (11). In the TmIGPS folding mechanism, the unfolded, U state initially collapses to an off-pathway intermediate, I_bp_, that must at least partially unfold to access the first on-pathway intermediate, I_a_. The conversion of I_a_ to the second on-pathway intermediate, I_b_, is the rate-limiting step in folding, rendering the subsequent faster I_b_ to N reaction as undetectable. The denaturant dependence of the ellipticity of I_a_, the dominant species after 10 s of folding, is coincident with that for I_bp_, demonstrating comparable stabilities for the off- and on-pathway intermediates (Fig. 1*C*). For unfolding, the N to I_b_ reaction is the minor fast phase between 2 and 4.5 M GdnHCl (Fig. 1*C*). The major slow unfolding phase corresponds to the rate-limiting conversion of I_b_ to I_a_. The N to N′ unfolding reaction, revealed by a rollover in the relaxation times at high denaturant concentrations, is a distinguishing feature of the TmIGPS mechanism. The very weak denaturant dependence of the N to N′ phase indicates a very small change in the buried surface area, justifying the N′ designation. As will be demonstrated in the HDX-MS experiment, I_eq_ is a composite of the I_a_ and I_bp_ species.

### HDX-MS Confirms and Expands the Folding Mechanism of TmIGPS.

#### Equilibrium experiments.

**Fig. 2.**
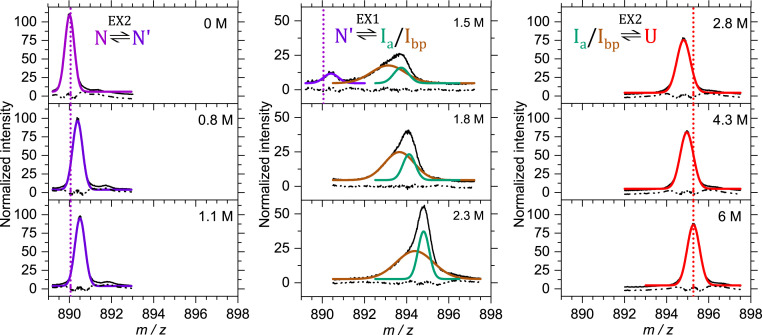
The results of pulse–quench H-to-D exchange of intact TmIGPS after a 9 d equilibration is displayed at selected GdnHCl concentrations. The normalized +28-charge state (solid black curves) was fitted with a pair of Gaussian functions to account for the presence of two intermediates (solid brown and green curves). The dotted lines show the deuterium uptake for native (violet) and unfolded (red) states. Residuals for the fitting curves are shown as dash-dot-dot black lines. The implied limits for the exchange reactions, EX1 and EX2, are indicated. A 10 s deuterium pulse was required to fully exchange the backbone NHs with the longest intrinsic exchange lifetime (∼3 s) as determined with program SPHERE (20).

**Fig. 3.**
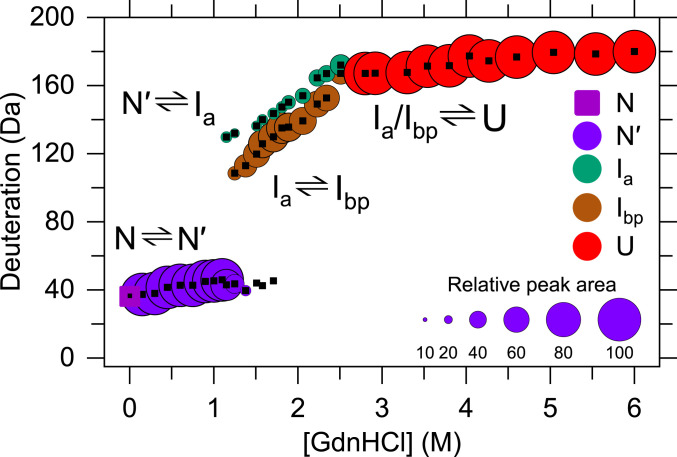
The deuterium uptake and relative peak areas of the +28-charge state are shown for species in the TmIGPS folding mechanism from equilibrium HDX-MS experiments on the intact protein after equilibration for 9 d in different GdnHCl concentrations. Gaussian fitting was used to determine the deuterium uptake (black dots) and the relative peak area (circles) for intermediates in the +28-charge state (Fig. 2). The maximum number of exchangeable NHs, 212 (222 residues, 9 prolines) sets an upper limit on the deuterium uptake. The uptake of 181 deuteriums in the unfolded control represent 15% back exchange with hydrogen during the workflow (*SI Appendix*, Table S2).

The CD and FL experiments were useful in defining a folding free energy surface for TmIGPS, but neither are capable of providing insights into the structures of the partially folded states on that surface. HDX-MS can provide a global assessment for the protection of backbone amide hydrogens (NHs) against exchange with solvent deuterium for the intact protein (20). The H-to-D exchange behavior of TmIGPS was monitored after equilibration at different GdnHCl concentrations (0 to 6 M) for 9 d. After equilibration, deuterium-labeled samples were quenched and loaded on a home-built HDX device and then detected by electrospray ionization mass spectrometry (ESI-MS) (*SI Appendix*, Fig. S3). The *m/z* peaks for the +28-charge state and the number of exchanged backbone NHs obtained after Gaussian fitting of ESI-MS data for the +28 charge state are shown (Figs. 2 and 3).

In the native state (N) at 0 M GdnHCl, TmIGPS has exchanged 34 NHs (*m/z* 890.0) with deuterium in comparison to its undeuterated state (*m/z* 888.8). The N peak shifts smoothly from *m/z* 890.0 (34 Da) to *m/z* 890.4 (46 Da) with increasing denaturant concentration, reflecting the transition from the N to the N′ state (Figs. 2 and 3). The N′ state disappears completely at ∼1.8 M GdnHCl, the same concentration reported by equilibrium FL experiment (Fig. 1*B*). At 1.2 M GdnHCl concentration, where N and I_eq_ are populated (Fig. 1*B*), a new peak appears at *m/z* 893.5 (130 Da) that grows in intensity as the N′ peak diminishes (Fig. 2). Further increases in GdnHCl concentration reveal the new peak to be a pair of overlapping peaks, a broad peak at *m/z* 892.7 (109 Da) and a narrow peak at *m/z* 893.6 (132 Da). The pair of peaks shift smoothly to higher *m/z* up to 2.3 M GdnHCl (*SI Appendix*, Table S2). At the same time, the peak area of the lower *m/z*, broader peak decreases as the area of the higher *m/z*, narrower peak increases (Fig. 3). By 2.8 M GdnHCl, where the U state is highly populated, only a single narrow peak is apparent (*m/z* 894.8, 167 Da). This peak shifts smoothly to a higher *m/z* up to 6 M GdnHCl (*m/z* 895.3 *m/z*, 180 Da).

#### Kinetic experiments.

The assignment of the pair of *m/z* peaks between 1.1 and 2.8 M GdnHCl was obtained by a kinetic HDX-MS experiment based on the TmIGPS folding mechanism (Scheme 1). After 10 s of refolding at 0.8 M GdnHCl, TmIGPS occupies only the I_a_ state, following escape from the I_bp_ trap and prior to the rate-limiting step in folding (*SI Appendix*, Fig. S4). A single peak with a narrow width is observed at *m/z* 893.0 in the kinetic refolding HDX-MS experiment, assigning the narrow peak to I_a_ and the broad peak to I_bp_ in the equilibrium HDX-MS experiment (*SI Appendix*, Table S3). The deuterium labeling of the I_b_ state was obtained by a kinetic unfolding HDX-MS experiment. TmIGPS was unfolded and pulse labeled with 1.5 M deuterated D_2_O/GdnHCl between 10 and 1,800 s (*SI Appendix*, Fig. S5*A*). The conversion of I_b_ to I_a_ at 1.5 M GdnHCl occurs with a time constant of ∼2.7 h (Fig. 1*C*), ensuring the kinetic unfolding HDX-MS data only reflected the protection against H-to-D exchange by I_b_ state. The N′ to I_b_ transition occurs with a time constant of ∼300 s (*SI Appendix*, Fig. S5*B*), consistent with the observed faster unfolding FL phase (Fig. 1*C*).

**Fig. 4.**
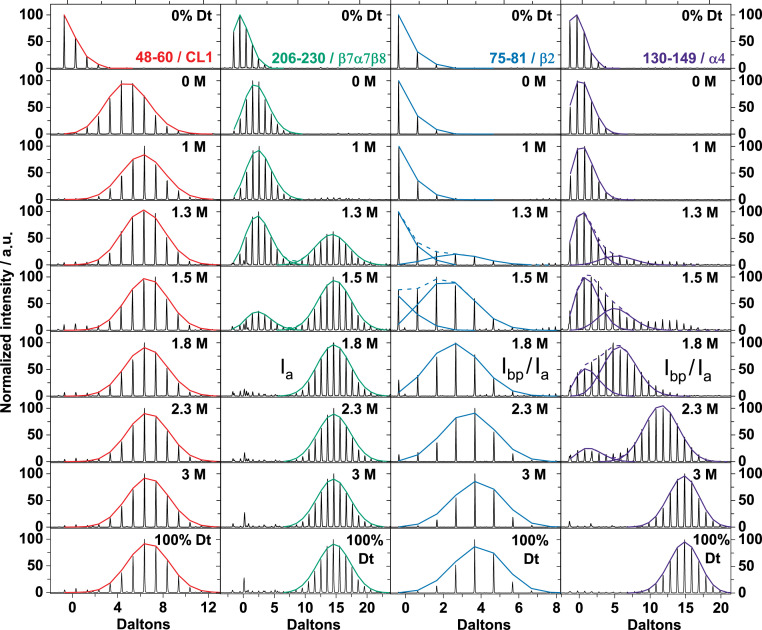
H-to-D exchange behavior and deuterium uptake of representative peptides from TmIGPS following proteolytic digestion of samples from equilibrium HDX-MS experiments at the indicated GdnHCl concentration. The raw ESI-MS spectra is shown, and the fitted isotopic envelope is colored according to the state from which exchange occurs. Peptide 48–60 (red) completely exchanges in the N′ state via an EX2 mechanism. Peptide 206–230 (green) completely exchanges in the I_a_ and/or I_bp_ states via an EX1 mechanism. Peptide 75–81 (blue) completely exchanges in the U state via an EX2 mechanism. Peptide 130–149 (violet) shows a late EX1 transition and completely exchanges in U state via an EX2 mechanism. The undeuterated and fully deuterated controls are shown in top and bottom frames, respectively.

Although the HDX-MS data shown in Figs. 2 and 3 were collected under equilibrium conditions, the results are informative about the limits of the kinetic processes that links various species on the TmIGPS free energy folding landscape. The exchange behavior of backbone NHs with deuterium is controlled by the rate constants for the opening (k_op_) and the closing (k_cl_) reactions that expose the backbone NH to solvent, and the intrinsic rate constant for exchange (k_ex_) of an exposed backbone NH under the experimental conditions (20). Under the EX1 limit, k_cl_ << k_ex_, and the exchange is controlled by k_op_. Under the EX2 limit, k_cl_ >> k_ex_, and exchange is controlled by k_op_/k_cl_ (i.e., the free energy difference between the open and closed states (∆G° = -RT x ln(k_op_/k_cl_)). At pH 7.2 and 25 °C, the average k_ex_ for amide hydrogens is ∼1 s^−1^ (20), defining EX1 processes as those with k_cl_ << 1 s^−1^ and EX2 processes as those with k_cl_ >> 1 s^−1^. The consequences on the HDX-MS data are that EX1 processes result in the coordinated peak area changes for the protonated and deuterated states, and EX2 processes result in the continuous change of the *m/z* value between the protonated and deuterated states. The assignment of the steps in the kinetic scheme for TmIGPS folding are shown (Scheme 1 and Fig. 2). EX2 processes reflect the undetected fast refolding of I_b_ to N′ and the N′ to N transitions. As expected, the very slow rate-limiting refolding step from I_a_ to I_b_ is controlled by an EX1 process that accounts for the simultaneous appearance of the N/N′ peak and the overlapping I_a_/I_bp_ peak between 1.2 and 1.7 M GdnHCl (Figs. 2–4). Surprisingly, another EX1 process links the high-energy states, I_a_ and I_bp_, between 1.2 and 2.5 M GdnHCl. The source of the structural differences between these two high-energy states and the I_b_ state can be determined by mapping the HDX protection on the amino acid sequence.

### Mapping the Structures of Folding Intermediates in TmIGPS with HDX-MS.

#### Equilibrium experiments.

HDX-MS results on the intact protein were useful in linking the folding intermediates in TmIGPS detected by equilibrium and kinetic optical experiments (CD and FL) and equilibrium HDX-MS experiments, but they cannot provide insights into their structures. Proteolytic digestion of the pulse-labeled protein from 0 to 5 M GdnHCl concentrations provides the desired information. The equilibration protocol, normalization controls, and deuterium-pulse labeling and quenching procedures were similar to that of the HDX-MS experiments on the intact protein (*SI Appendix*, Fig. S6). After quenching, TmIGPS samples were proteolytically digested on a chilled online digestion pepsin column. The resulting peptides were separated by ultra-pressure liquid chromatography (UPLC) and analyzed by ESI-MS. A total of 70 overlapping peptides, covering 97% of the TmIGPS amino acid sequence, were analyzed (*SI Appendix*, Fig. S7).

**Fig. 5.**
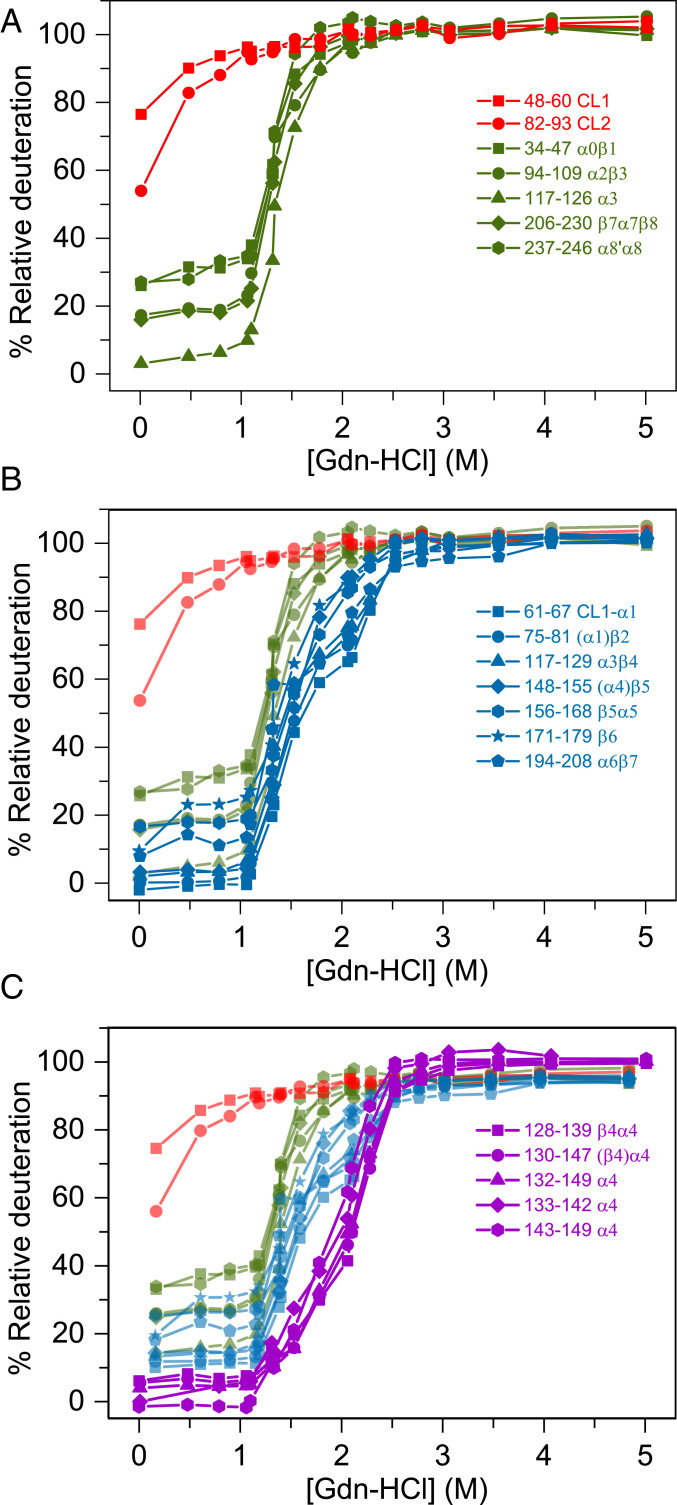
TmIGPS peptides derived from equilibrium HDX-MS experiments (0 to 5 M GdnHCl) were divided into different classes based on their H-to-D exchange behavior. (*A*) Class I (red) and Class II (green) peptides represent segments that exchange in the N and/or N′ states or the I_a_ and/or I_bp_ states, respectively. (*B*) Class III (blue) peptides represent segments where 15 to 40% of the backbone NHs are protected in I_a_ and/or I_bp_ and exchange out in the U state via an EX2 mechanism. (*C*) Class IV (violet) peptides represent segments where 45 to 60% of the NHs are protected against HDX in I_a_ and/or I_bp_ and exchange out in U via an EX2 mechanism. Only the peptides spanning residues 128 to 149 (β4α4) show this behavior. Secondary structures are marked in parenthesis if those peptides have only one or two amino acids on their N termini (*SI Appendix*, Fig. S7). Experiments were repeated twice with separate titrations and analyzed individually. Results are shown for one of the titrations.

The peptides were sorted into four different classes based on their H-to-D exchange mechanism and protection in intermediates. Representative spectra (Fig. 4) and titration curves (Fig. 5) for the selected set of peptides are shown. The fitted isotopic envelope of representative peptides at every GdnHCl concentration is shown in *SI Appendix*, Dataset S1.

Class I: The majority of the backbone NHs are exchanged within the 10 s deuterium pulse at 0 M GdnHCl. The remaining protection is lost via an EX2 mechanism with the formation of the N′ state at 1.1 M GdnHCl (Fig. 5*A*).
Class II: After the initial rapid exchange of a few backbone NHs via an EX2 mechanism, these peptides are protected in N′ and exchange their remaining backbone NHs via an EX1 mechanism with I_a_ and/or I_bp_ (Fig. 5*A*).
Class III: 15 to 40% of the backbone NHs in these peptides are protected in I_a_ and/or I_bp_ and exchange out in the U state via an EX2 mechanism (Fig. 5*B*).
Class IV: 45 to 60% of the NHs are protected against HDX in I_a_ and/or I_bp_ and exchange out in U via an EX2 mechanism for peptides spanning residues 128 to 149 (β4α4) (Fig. 5*C*). Compared to the Class III peptides, the higher apparent midpoint for the first transition in uptake implies a stronger protection for β4α4.

**Fig. 6.**
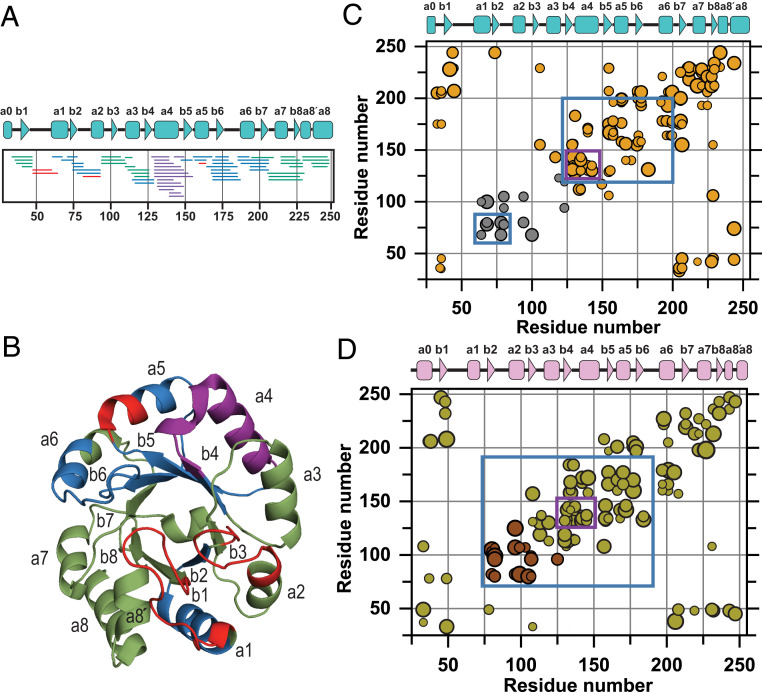
Cores of stability in high-energy states are similar in evolutionarily distinct orthologs of IGPS. (*A*) Peptides and (*B*) ribbon diagram of TmIGPS are colored based on their protection pattern in I_a_ and I_bp_ states (Figs. 4 and 5): None (green), weak to moderate (blue), strong (violet), and segments which completely exchange in N′ (red). (*C*) Protection against exchange in TmIGPS and (*D*) SsIGPS folding intermediate(s) is displayed on their 2D ILV contact map (PDBs:1I4N and 2C3Z). The colors of the filled symbols represent different ILV clusters. The purple box in segment from residues 126 to 150 in both maps contains the most strongly protected segment and represents the structured region in the I_bp_ and/or I_a_ states. The blue box contains weak-to-moderate protection and surrounds the stability cores in I_a_ and I_bp_ states. Contacts made between any two ILV residues are represented by participating partners on the *x*- and *y*-axes. The surface area buried by the partner ILV residues is proportional to the size of the circle. The (βα)_8_ TIM barrel motif of TmIGPS (cyan) and SsIGPS (pink) is presented above *C* and *D*. The SsIGPS protection pattern was taken from ref. 15, with permission from Elsevier.

The four classes of peptides are shown in Fig. 6*A* and mapped onto the ribbon diagram of TmIGPS in Fig. 6*B*. The protection against exchange in I_a_ and/or I_bp_ is located in the α1β2 and β5α5β6α6β7 segments (Class III) and the β4α4 segment (Class IV). These regions, when mapped on a two-dimensional (2D) contact plot of isoleucine, leucine, and valine (ILV) side chains (Fig. 6*C*), show a strong correlation between clusters of ILV side chains and protection against exchange in these intermediates. Notably, the β4α4 segment has the highest density of ILV contacts. A similar correlation has been observed previously for SsIGPS [Fig. 6*D* (15)], emphasizing a key role for hydrophobicity in stabilizing folding intermediates. The absence of protection in β1 and β8 for I_a_ and I_bp_ means that the β-barrel has opened via the rate-limiting step from I_b_ to I_a_. Consequently, the significant density of ILV contacts found in the 2D maps for these regions in the native state does not exist in I_a_ and I_bp_ intermediates.

#### Kinetic experiments.

The protection patterns in the individual N and N′ states could be mapped at equilibrium at 0 and 1.1 M GdnHCl. Kinetic experiments were required to isolate the I_a_ and I_b_ species and map their HDX protection patterns (see above). The peptides from N′ uniformly exhibit greater deuteration (i.e., more exposed to solvent) than their counterparts in the N state, suggesting a general loosening of the structure (*SI Appendix*, Fig. S8 *A* and *B*). I_b_ has a closed β-barrel and a nearly intact α-helical shell (*SI Appendix*, Fig. S9 *A* and *B*). The segments spanning α2β3α3 and α8′α8, however, experience significantly greater exchange, indicating them as the principal sites of annealing to reach the N state. In the mixture of I_a_ and I_bp_ at equilibrium, the β-barrel is open and only the α1β2 and the β4α4β5α5β6α6β7 segments offer protection against exchange (Class III and Class IV peptides) (Fig. 6*B* and *SI Appendix*, Fig. S8*C*). These segments in TmIGPS also contain βα-hairpin clamps (*SI Appendix*, Fig. S10) known to stabilize TIM barrel proteins (21, 22). Although aggregation in stopped-flow refolding experiments precluded a direct structural analysis of I_bp_, peptide mapping of I_a_ revealed strong protection against exchange in β2 and β4α4β5 under strongly folding conditions (*SI Appendix*, Fig. S9*C*). The comparison of I_a_ with the mixture of I_a_ and I_bp_ at equilibrium in 1.8 M GdnHCl shows that I_bp_ also protects α1 and α5β6α6β7. We note that the lower mean *m/z* value for I_bp_ (i.e., less deuterated/more protected) is accompanied by a broader distribution than seen for the N, N′, I_a_, and U states (Fig. 2). I_bp_ appears to be stabilized by a structurally different ensemble than its counterparts in the folding mechanism.

### Bioinformatic Analysis of Hydrophobicity in the IGPS Family of TIM Barrels across Evolution.

The striking correlation between the HDX protection patterns in bacterial TmIGPS and archaeal SsIGPS, especially the strongest protection in the β4α4 segment (Fig. 6 *C* and *D*), motivated a bioinformatics analysis of the hydrophobicity of thousands of sequences of IGPS orthologs from the bacterial, acrchaeal, and eukarya superkingdoms. We used the Kyte–Doolittle hydropathy score (23) and a rolling five-residue window to calculate the hydrophobicity in members of a well distributed and large IGPS TIM barrel family. The Kyte–Doolittle hydrophobicity scale was chosen as it closely mimics the dominant role of ILV side chains reflected in Fig. 6 *C* and *D*.

**Fig. 7.**
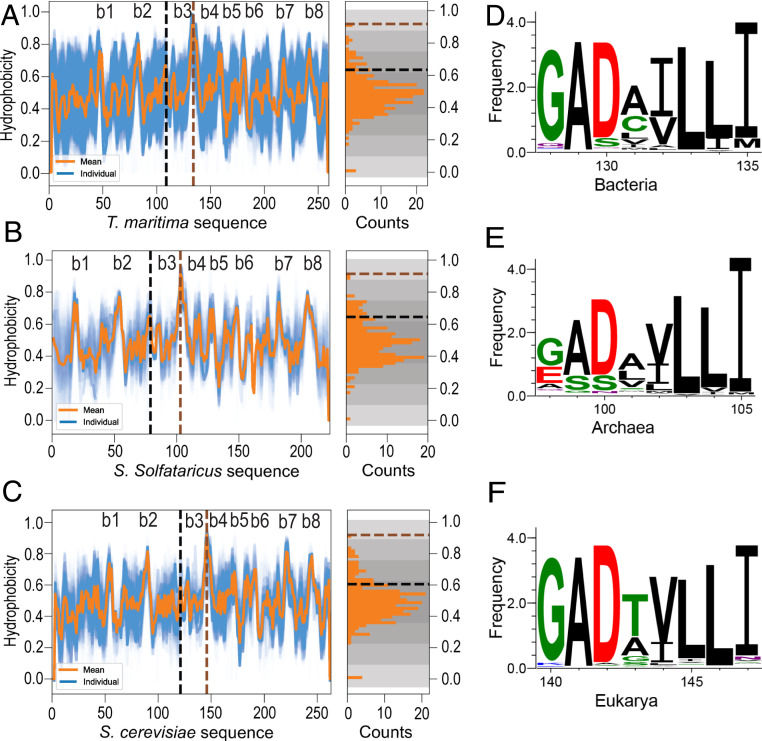
Hydrophobicity patterns and sequence logos for IGPS TIM barrels from the three superkingdoms. (*A*) Bacteria, 5,808 sequences; (*B*) Archaea, 279 sequences; and (*C*) Eukarya, 720 sequences. The Kyte–Doolittle hydrophobicity of a five-residue window for individual sequences (cyan) is shown along with the mean hydrophobicity of all sequences (orange). The sequence numbers correspond to the IGPS sequences from the indicated organism (below the image). The β4 strand in all three superkingdoms has highest hydrophobicity and is >3 SDs above the mean (brown dashed line in the counts profile). The hydrophobicity of the β3 strand (black dashed line in the counts profile) is lowest among all eight β-strands and lies closer to mean value. (*D*–*F*) Logos of the sequences preceding and including β4 for the corresponding superkingdom. The glycine-alanine-aspartic acid (GAD) sequence is highly conserved and corresponds to its role in formation the βα-hairpin clamp with β3. The exceedingly high hydrophobicity of β4 reflects the almost exclusive presence of branched aliphatic side chains.

The observed patterns reflect the periodicity of the hydrophobic β-strands, as expected for the (βα)_1–8_ TIM barrel architecture (Fig. 7 *A*–*C*). The site of highest hydrophobicity is strongly conserved in the β4 strand, and its score is >3 SDs higher than the mean hydrophobicity for each of the three superkingdoms. Strikingly, the hydrophobicity score for the adjacent β3 strand is the lowest of the eight β-strands and close to mean for the entire sequence. The sequence logos show that ILV residues are highly favored in the β4 strand for all three superkingdoms (Fig. 7 *D*–*F*), demonstrating a defining role for the branched aliphatic side chains in the folding of the IGPS family of proteins that spans over a billion years of evolution (*SI Appendix*, Fig. S11).

Although branched aliphatic side chains are often associated with β-strands, leucine favors α-helices. The β4 strand shows strong conservation for consecutive leucine residues at equivalent positions in β4 for all three superkingdoms (*SI Appendix*, Fig. S12). The net effect for the sequences corresponding to β4 is a surprising tendency toward α-helix formation (*SI Appendix*, Fig. S13). The sequence logo for β3 shows a strong preference for valine/isoleucine at the first position and leucine at the second (*SI Appendix*, Fig. S14). The preference for arginine and lysine at the third and fourth positions accounts for the low hydrophobicity of β3 (Fig. 7). K110 (β3) is absolutely conserved across all members of the IGPS class of enzymes because it plays a key role in the active site architecture (17). A final surprise in the bioinformatics analysis was the strong conservation of the glycine-alanine-aspartic acid (GAD) βα-hairpin clamp linking β3 and β4 in all three superkingdoms (Fig. 7 *D*–*F* and *SI Appendix*, Fig. S12). A previous saturation mutagenesis analysis of this region in SsIGPS, TmIGPS, and TtIGPS revealed a long-range, allosteric connection between the βα-hairpin clamp and the enzymatic active site at the opposite end of the β-barrel (17). Taken together, the conservation of these sequence features reflects essential and orthogonal roles in the folding and function for the IGPS family of proteins.

## Discussion

### Structure of Folding Intermediates in TmIGPS.

The equilibrium and kinetic HDX-MS experiments on TmIGPS revealed structural insights for all of the partially folded states on the folding free energy surface (Fig. 6*B* and *SI Appendix*, Figs. S8 and S9). The N′ state maintains its TIM barrel fold, with fraying at the ends of α-helices and β-strands accounting for its reduced protection. The I_b_ state has a closed β-barrel; however, the α2β3α3 and α8′α8 segments framing the active site become dynamic and exchange their amide hydrogens with deuterium. Although the β-barrel is open in both the I_a_ and I_bp_ states, I_a_ only offers strong protection in β2 and β4α4β5. We were not able to directly assess the protection of I_bp_ because the protein aggregates under stopped-flow conditions. However, the simultaneous presence of I_a_ and I_bp_ at equilibrium implies that I_bp_ also offers protection in α1β2 and β4α4β5α5β6α6β7. Despite the lack of a direct contact between the sequence-separated protected segments in the I_a_ and I_bp_ states, the coincident loss in protection at increasing GdnHCl concentrations (Fig. 5 *B* and *C*), is evidence for their cooperative transition to the unfolded state. However, the uniquely strong, Class IV, protection of the β4α4 segment observed in the equilibrium experiments (Figs. 4 and 5 *B* and *C*) shows that the β4α4β5 and/or β4α4β5α5β6α6β7 modules do not unfold in a two-state fashion. The selective protection of a single βα element in a structure with eightfold βα symmetry highlights the role of sequence in protection against exchange.

We have earlier proposed that large clusters formed by aliphatic side chains of ILV inhibit water penetration and hydrogen exchange in partially folded states and form cores of stability in other βα proteins (14, 24, 25). The 2D contact map for ILV side chains in the bacterial TmIGPS reveals two ILV clusters, markedly similar to its evolutionarily distinct ortholog, archaeal SsIGPS (Fig. 6 *C* and *D*) (15). In both cases, a large cluster is primarily located in the C-terminal half of the barrel, highly dense in the β4α4 region, and includes a few contacts that link the N and C termini. For TmIGPS, a small cluster is localized in the N-terminal half of the barrel and spans the α1β2α2β3α3 segments. A corresponding cluster in SsIGPS spans β2α2β3α3. For both TmIGPS and SsIGPS, side chain–main chain hydrogen bonds between even numbered β-strands and their preceding odd-numbered counterparts, β1β2 and β3β4, form stabilizing βα-hairpin clamps (21). In TmIGPS, the core of stability in intermediates (I_a_ and I_bp_) is defined by protection against exchange in a small (α1β2) and a large cluster (β4α4β5α5β6α6β7). The strongest protection is in the β4α4 module that has the highest density of ILV contacts in the folded TmIGPS structure. These observations are strikingly similar to those for the archaeal ortholog SsIGPS (15, 16) and make a strong case for the Branched Aliphatic Side Chains hypothesis (BASiC) as a major determinant of TIM barrel folding reactions (24).

### A Conserved Nonnative Folding Nucleus in the IGPS TIM Barrels?

The exceedingly high hydrophobicity and predicted helix propensity of β4 in the IGPS family of TIM barrels (Fig. 7 *A*–*C* and *SI Appendix*, Figs. S12 and S13) may provide an explanation for the puzzling observation of the uniquely strong, Class IV, protection for the segment corresponding to β4α4 in the native structure. How could a single β-strand offer protection against exchange in the absence of its adjacent partners, β3 and/or β5? Taken together, these bioinformatics and experimental observations may find a common explanation in the formation of a helical hairpin from β4 and α4 in I_bp_ for both TmIGPS and SsIGPS. We speculate that the 100’s of nanoseconds folding dynamics of α-helices would allow the β4 and α4 segments to sample helical structures that could rapidly associate into a helical hairpin. The hairpin would offer protection against exchange in nascent β4 and α4 via intrahelical H-bonds and be stabilized by the formation of a nonnative ILV hydrophobic cluster between the side chains of the β4 and α4 segments. The hairpin would appear in <ms and be sufficiently stable to drive the formation of I_bp_. This putative nonnative structure could serve as a nucleus to recruit adjacent elements under folding conditions and offer protection against exchange for the α5β6α6β7 segment. Productive folding would require back-tracking to disrupt I_bp_ and enable the formation of a native-like β4/α4/β5 trio in I_a_, where β4 and β5 would offer mutual protection against exchange. The broad distribution of protection against exchange in I_bp_ (Fig. 2), in the context of the narrower distributions for N, N′, I_a_, and U, implies an alternative structural ensemble, possibly a molten globule stabilized by the hydrophobic effect (26, 27). Although aggregation precluded direct measurement of protection in I_bp_, the remarkable resistance to exchange for β4α4 in the presence of high concentrations of GdnHCl (Figs. 4 and 5) and its local connectivity make a strong argument for its independent and early formation of a folding nucleus that drives the folding of both I_a_ and I_bp_.

The conjecture that β-strands might initially fold as α-helices is consistent with previous HDX studies of the alpha subunit of Trp synthase (αTS), also a TIM barrel, RNase H, an α + β protein, and β-lactoglobulin (βLG), which predominantly contains β structure. An HDX-NMR study on αTS found strong protection in a continuous string of five residues, rich in ILVs, for a pair of adjacent β-strand segments (28), and a β-strand and adjacent α-helix was the first segment to be protected in the refolding of RNase H (29). A complementary FL refolding study of RNase H revealed nonnative structure in the microsecond time range, consistent with an early misfolding reaction (30). The transient helix formation in the early folding intermediate of βLG was first detected by the increased levels of nonnative α-helical CD signal on the millisecond time scale (31, 32). Later, ultra-rapid mixing techniques in conjunction with Trp fluorescence and HDX-NMR were used to characterize the structural and dynamic properties of partially helical compact state of early refolding intermediate in βLG (33).

### Why Conserve a Nonnative Helical Hairpin and the Preceding βα-Hairpin Clamp?

It is striking that we observed the same folding mechanism and folding structural elements for TIM barrels from both archaea and bacteria, which diverged nearly ∼4 billion y ago. Further, the sequence signature for this biophysical feature, the conserved ILLI motif in β4, has been maintained for billions of years of evolution in archaea, bacteria, and eukaryotes. This observation provides strong evidence that the folding mechanism of IGPS TIM barrels first appeared in the last universal common ancestor of these ancient proteins and has persisted for billions of years.

The sequence density of ILV residues in the β4 segment is responsible for its extreme hydrophobicity. If not protected by a rapidly forming local structure, even if nonnative, it could nucleate by intermolecular interactions leading to aggregation. However, over ∼4 billion y, it might have been expected to either evolve to a less hydrophobic sequence and/or surrender its primary nucleation role in folding to another βα element. The answer may be that this putative nonnative structure equilibrates within seconds with the on-pathway, I_a_, intermediate on the productive path to the native state. Synthesis on ribosome is slower, 5 to 20 aa/sec (34), allowing sufficient time for the sequence to escape the kinetic trap in I_bp_ as the protein extrudes from the tunnel and encounters the trigger factor chaperone. In addition, the helical propensity of the β4 sequence in I_bp_ may protect this very hydrophobic sequence from undergoing intermolecular interactions that can lead to aggregation or the formation of amyloid fibers (33). In another scenario, the transient helical structure formed by the β4 sequence in I_bp_ might be essential to disfavor the nonnative pairing of β4 with other β-strands early in folding. Thus, the β4 sequence would not experience an evolutionary pressure to eliminate a nonnative structure. Once this initial step on the folding pathway was established, it may have constrained the further evolution of the sequence without introducing off-pathway or misfolded intermediates. If correct, the very high conservation of the hydrophobic sequences for β4 in all three superkingdoms implies a set of mutations that became fixed in the LUCA and continues to dictate the initial events in the folding of the IGPS family of TIM barrel proteins. It would be interesting to know if TIM barrel paralogues have different nucleation sites that also persist over evolutionary time. For example, *Escherichia coli* αTS protects β2 and β3 against exchange in a high-energy state (28). Is it the case that once a strong nucleation sequence appears in TIM barrels, it becomes fixed throughout evolutionary time? Further experiments are required to answer this question.

The reason for the strong conservation of the βα-hairpin clamp may find its explanation at the final stage of folding when the native conformation appears, as observed in a previous mutational analysis of a similar βα-hairpin clamp in αTS (21). The βα-hairpin clamp stabilizes its rate-limiting transition state and the native conformation. However, it is intriguing to speculate that the βα-hairpin clamp may also play a transient role early in folding by colocating the pair of branched aliphatic side chain amino acids at the N terminus of β3 with the ILV-rich β4 segment proposed to form a helical pair with α4. The putative early and known late roles in folding for the GAD sequence may explain its very high conservation.

### IGPS Folding Free Energy Surfaces: Landscapes or Foldons?

From the perspective of polymer physics and statistical mechanics, Zwanzig (35) long ago pointed out that the rapid formation of local biases toward the native structure in an unfolded polypeptide chain, when coupled with their assembly in a myriad of ways into higher order structures, are sufficient to drive folding reactions that occur well within a biological time frame. Landscape Theory (36) built on that observation describes a funnel-like energy landscape that would allow for many possible pathways to proceed from the unfolded manifold of microstates to the native state. Concurrent with this development were the experiments of Englander and colleagues (37) that were interpreted in terms of the progressive development of native structure by the sequential formation of higher-order structure by the ordered assembly of simple elements of structure referred to as foldons. In effect, the foldon concept defined a tightly proscribed pathway from the unfolded state to the native state. A lively debate about these two diametrically opposed views of folding reactions continues to the present (38–40).

The folding mechanism of the IGPS family of TIM barrels proteins is not well described by either the Landscape Theory or the Foldon Model. The eightfold βα symmetry does not result in eight comparable folding modules to initiate folding, as might be expected from the simplest view of Landscape Theory. Rather, the (βα)_4_ module is highly favored after only a few microseconds, comparable to the folding times of small proteins and domains from larger proteins (41). In effect, the funnel narrows considerably to reach the I_bp_ state, a transient species that matures through a series of subsequent steps to reach the native conformation. Surprisingly, the peculiar details of the sequence appear to result in the formation of a nonnative structure that must at least partially unfold to allow access to the productive folding pathway. The structure of the subsequent on-pathway intermediate involves elements from several βα modules, likely stabilized by a cluster of branched aliphatic side chains. As reported in the present study, these intermediates are largely conserved across the bacterial and archaeal superkingdoms, speaking to a robust folding pathway with a defined set of partially folded states whose structures appear to be stabilized by the hydrophobic effect. If the Foldon Model is operative, it must exert its effects on an ensemble of microstates within the energy wells of these intermediates, as a pseudoequilibrium prior to the transition to the next state on the folding pathway. For both I_a_ and I_bp_ intermediates, the sequence corresponding to (βα)_4_ segment is the core of stability around which adjacent elements of structure condense.

### Perspective.

The conservation of the folding mechanism for a pair of IGPS TIM barrels from bacterial and archaeal organisms reflects the conservation of the structures of off- and on-pathway intermediates across evolutionary time. Although the examined sequences are only 30% identical, the active site residues and key elements of the sequence are very highly conserved. The conserved folding elements arose in the LUCA and, in the absence of selective pressure, have become fixed and define this family of TIM barrel proteins. We speculate that other TIM barrel families have conserved but different nucleation sites that are also rich in sequence-local ILV residues [e.g., αTS (28, 42)]. Examination of their sequences might not only provide insight into their early folding events but also be a fingerprint distinguishing various families of this ubiquitous fold.

The de novo design of TIM barrels, a quest for >25 y (43, 44), has thus far relied on tethering identical (βα)_1–4_ halves (45) or four repeating βαβα units (43). The asymmetry observed in the highly favored aliphatic sequences for (βα)_4_ in IGPS TIM barrels suggests that design algorithms might benefit from the lessons of nature to achieve efficient and rapid folding while avoiding aggregation.

## Materials and Methods

### Protein Expression and Purification.

Recombinant TmIGPS with ∆1–31 deletion corresponding to helix-00 and Cys101Ser mutation in the crystal structure [pdb 1I4N (46)] was expressed in *E. coli* strain BL21 Codonplus (DE3)RIL. TmIGPS without His6-tag was purified by using TEV protease and a series of chromatographic steps. TmIGPS purity was confirmed (> 98%) with SDS PAGE and ESI-MS measurement on a Synapt G2-Si (Waters Corporation, Milford, MA) quadrupole time-of-flight Q-TOF ESI mass spectrometer.

### Equilibrium and Kinetic Folding Studies with CD and FL Spectroscopy.

CD and tryptophan fluorescence experiments were done for a range of GdnHCl concentrations at pH 7.2 and 25 °C. The buffer in all experiments contained 10 mM potassium phosphate and 10 mM KCl. Far-UV CD data at steady state were collected from 260 nm to 200 nm by using a quartz cuvette of 5 mm pathlength. Three replicate CD spectra were collected and averaged. The equilibrium emission spectra after excitation at 280 nm were collected between 300 and 450 nm at a 1 nm interval and averaged over three traces. Manual mixing was used to initiate slow unfolding and refolding kinetics of TmIGPS. The change in ellipticity as a function of time was monitored at 222 nm in a quartz cuvette of 5 mm pathlength. The time dependent change in fluorescence emission spectra at 320 nm was measured after excitation at 280 nm. The dead-time of the manual mixing experiments was 3 s, and the instrument response time was about 5 s. The fast unfolding and refolding kinetics measurements were monitored with stopped-flow instruments. CD data were collected at 222 nm with a dead time of 5 ms. Stopped-flow fluorescence experiments were performed with a dead-time of 2 ms. The excitation wavelength was 280 nm while the emission was monitored using a 320 nm cutoff filter.

### Intact HDX-MS Experiments.

The H-to-D exchange behavior of intact TmIGPS was monitored after equilibration for 9 d at different GdnHCl concentrations. After equilibration, we applied a 1:20 pulse of deuterated D_2_O/GdnHCl at the same GdnHCl concentration for 10 s at pD 7.2 and 25 °C. The ∼95% deuterated solution was quenched by a 1:5 dilution with 200 mM potassium phosphate on ice to reduce the pH to 2.5. Small volume of ice cold protonated 7 M GdnHCl at pH 2.5 (0.2% formic acid) was added in quenched samples so that all the samples had ∼1 M GdnHCl before loading. The 50 µl quenched samples containing ∼620 ng intact TmIGPS were injected manually on a home built HDX module. Chromatographic separations were performed using a Waters Acquity UPLC fitted with a Waters C4 BEH (300Å, 1.7 µm, 2.1 mm × 50 mm) column interfaced to a Waters Synapt G2-Si ESI mass spectrometer operating in the positive ion electrospray mode. Three blank LC-MS runs with 50% isopropanol injection were used to minimize the carry over between TmIGPS samples. OriginPro and Savuka softwares were used for manual data analysis.

### Peptide Level HDX-MS Experiments.

The GdnHCl equilibration for 9 d followed by pulse labeling (1:12) and quenching steps (1:5) for peptide level experiments were similar as intact level experiments. 50 µl quenched samples containing ∼800 ng intact TmIGPS were injected manually on a home built HDX module, where TmIGPS was digested in a cooled online immobilized pepsin column (Waters Enzymate BEH 300 Å, 5 µm, 2.1 mm × 30 mm). Cleaved peptides were trapped on a Waters C18 BEH VanGuard precolumn (300 Å, 1.7 µm, 2.1 mm × 5 mm) and separated using a Waters C18 BEH (300 Å, 1.7 µm, 1 mm × 100 mm) column using the Waters Acquity UPLC-Synapt G2-Si interface as described above. Three blank LC-MS runs with 50% isopropanol injection were used to minimize the carry over between TmIGPS samples. The generation of peptide list was automated (Waters PLGS) while the search, validation and fitting of peptides in HDX-MS experiments was semiautomated [ExMS2 (47) and HX-Express (48)].

### Bioinformatics Analysis of IGPS Family of TIM Barrels.

IGPS amino acid sequences were downloaded from Pfam database (49) (id: PF00218) and sequences with >95% identity were culled. Sequences from each superkingdom were aligned separately. The gaps were removed from each sequence and hydrophobicity was calculated on the Kyte-Doolittle scale with a 5-residue rolling window. The hydrophobicity values were plotted for positions in the alignment that correspond to the reference sequence used for each superkingdom. The mean hydrophobicity for each position was calculated and plotted along with SD. Crystal structures of TmIGPS [PDB:1i4n (46)] and SsIGPS [PDB:2C3Z (50)] were used as reference to follow the secondary structures for bacterial and archaeal sequences. Sequence logos were generated from aligned sequences and using online server WebLogo3 (51). Secondary structures were predicted by online server JPred4 (52).

## Supplementary Material

Supplementary File

Supplementary File

## Data Availability

All study data are included in the article and/or supporting information.

## References

[1] R. L. Baldwin, Intermediates in protein folding reactions and the mechanism of protein folding. Annu. Rev. Biochem. 44, 453–475 (1975). 1094916 10.1146/annurev.bi.44.070175.002321

[2] K. A. Scott, V. Daggett, Folding mechanisms of proteins with high sequence identity but different folds. Biochemistry 46, 1545–1556 (2007). 17279619 10.1021/bi061904l

[3] M. S. Newton, V. L. Arcus, M. L. Gerth, W. M. Patrick, Enzyme evolution: Innovation is easy, optimization is complicated. Curr. Opin. Struct. Biol. 48, 110–116 (2018). 29207314 10.1016/j.sbi.2017.11.007

[4] A. A. Nickson, J. Clarke, What lessons can be learned from studying the folding of homologous proteins? Methods 52, 38–50 (2010). 20570731 10.1016/j.ymeth.2010.06.003 PMC2965948

[5] R. G. Smock, I. Yadid, O. Dym, J. Clarke, D. S. Tawfik, De novo evolutionary emergence of a symmetrical protein is shaped by folding constraints. Cell 164, 476–486 (2016). 26806127 10.1016/j.cell.2015.12.024 PMC4735018

[6] C.-I. Brändén, The TIM barrel—the most frequently occurring folding motif in proteins: Current Opinion in Structural Biology 1991, 1:978–983. Curr. Opin. Struct. Biol. 1, 978–983 (1991).

[7] N. Nagano, C. A. Orengo, J. M. Thornton, One fold with many functions: The evolutionary relationships between TIM barrel families based on their sequences, structures and functions. J. Mol. Biol. 321, 741–765 (2002). 12206759 10.1016/s0022-2836(02)00649-6

[8] L. Carstensen., Conservation of the folding mechanism between designed primordial (βα)8-barrel proteins and their modern descendant. J. Am. Chem. Soc. 134, 12786–12791 (2012). 22758610 10.1021/ja304951v

[9] A. D. Goldman, R. Samudrala, J. A. Baross, The evolution and functional repertoire of translation proteins following the origin of life. Biol. Direct 5, 15 (2010). 20377891 10.1186/1745-6150-5-15 PMC2873265

[10] B. Reisinger., Evidence for the existence of elaborate enzyme complexes in the Paleoarchean era. J. Am. Chem. Soc. 136, 122–129 (2014). 24364418 10.1021/ja4115677

[11] W. R. Forsyth, O. Bilsel, Z. Gu, C. R. Matthews, Topology and sequence in the folding of a TIM barrel protein: Global analysis highlights partitioning between transient off-pathway and stable on-pathway folding intermediates in the complex folding mechanism of a (betaalpha)8 barrel of unknown function from B. Subtilis. J. Mol. Biol. 372, 236–253 (2007). 17619021 10.1016/j.jmb.2007.06.018

[12] O. Bilsel, J. A. Zitzewitz, K. E. Bowers, C. R. Matthews, Folding mechanism of the alpha-subunit of tryptophan synthase, an alpha/beta barrel protein: Global analysis highlights the interconversion of multiple native, intermediate, and unfolded forms through parallel channels. Biochemistry 38, 1018–1029 (1999). 9893998 10.1021/bi982365q

[13] W. R. Forsyth, C. R. Matthews, Folding mechanism of indole-3-glycerol phosphate synthase from sulfolobus solfataricus: A test of the conservation of folding mechanisms hypothesis in (beta(alpha))(8) barrels. J. Mol. Biol. 320, 1119–1133 (2002). 12126630 10.1016/s0022-2836(02)00557-0

[14] B. N. Gangadhara, J. M. Laine, S. V. Kathuria, F. Massi, C. R. Matthews, Clusters of branched aliphatic side chains serve as cores of stability in the native state of the HisF TIM barrel protein. J. Mol. Biol. 425, 1065–1081 (2013). 23333740 10.1016/j.jmb.2013.01.002 PMC3696590

[15] Z. Gu, J. A. Zitzewitz, C. R. Matthews, Mapping the structure of folding cores in TIM barrel proteins by hydrogen exchange mass spectrometry: The roles of motif and sequence for the indole-3-glycerol phosphate synthase from sulfolobus solfataricus. J. Mol. Biol. 368, 582–594 (2007). 17359995 10.1016/j.jmb.2007.02.027 PMC2040069

[16] Z. Gu, M. K. Rao, W. R. Forsyth, J. M. Finke, C. R. Matthews, Structural analysis of kinetic folding intermediates for a TIM barrel protein, indole-3-glycerol phosphate synthase, by hydrogen exchange mass spectrometry and Gō model simulation. J. Mol. Biol. 374, 528–546 (2007). 17942114 10.1016/j.jmb.2007.09.024 PMC2735044

[17] Y. H. Chan, S. V. Venev, K. B. Zeldovich, C. R. Matthews, Correlation of fitness landscapes from three orthologous TIM barrels originates from sequence and structure constraints. Nat. Commun. 8, 14614 (2017). 28262665 10.1038/ncomms14614 PMC5343507

[18] I. S. Povolotskaya, F. A. Kondrashov, Sequence space and the ongoing expansion of the protein universe. Nature 465, 922–926 (2010). 20485343 10.1038/nature09105

[19] C. L. Worth, S. Gong, T. L. Blundell, Structural and functional constraints in the evolution of protein families. Nat. Rev. Mol. Cell Biol. 10, 709–720 (2009). 19756040 10.1038/nrm2762

[20] Y. Bai, J. S. Milne, L. Mayne, S. W. Englander, Primary structure effects on peptide group hydrogen exchange. Proteins 17, 75–86 (1993). 8234246 10.1002/prot.340170110 PMC3438223

[21] X. Yang, S. V. Kathuria, R. Vadrevu, C. R. Matthews, Betaalpha-hairpin clamps brace betaalphabeta modules and can make substantive contributions to the stability of TIM barrel proteins. PLoS One 4, e7179 (2009). 19787060 10.1371/journal.pone.0007179 PMC2747017

[22] X. Yang, R. Vadrevu, Y. Wu, C. R. Matthews, Long-range side-chain-main-chain interactions play crucial roles in stabilizing the (betaalpha)8 barrel motif of the alpha subunit of tryptophan synthase. Protein Sci. 16, 1398–1409 (2007). 17586773 10.1110/ps.062704507 PMC2206699

[23] J. Kyte, R. F. Doolittle, A simple method for displaying the hydropathic character of a protein. J. Mol. Biol. 157, 105–132 (1982). 7108955 10.1016/0022-2836(82)90515-0

[24] S. V. Kathuria, Y. H. Chan, R. P. Nobrega, A. Ozen, C. R. Matthews, Clusters of isoleucine, leucine, and valine side chains define cores of stability in high-energy states of globular proteins: Sequence determinants of structure and stability. Protein Sci. 25, 662–675 (2016). 26660714 10.1002/pro.2860 PMC4815418

[25] A. Radzicka, R. Wolfenden, Comparing the polarities of the amino acids: Side-chain distribution coefficients between the vapor phase, cyclohexane, 1-octanol, and neutral aqueous solution. Biochemistry 27, 1664–1670 (1988).

[26] T. Okabe, S. Tsukamoto, K. Fujiwara, N. Shibayama, M. Ikeguchi, Delineation of solution burst-phase protein folding events by encapsulating the proteins in silica gels. Biochemistry 53, 3858–3866 (2014). 24867232 10.1021/bi5003647

[27] K. Kuwajima, The molten globule, and two-state vs. Non-Two-State folding of globular proteins. Biomolecules 10, 407 (2020). 10.3390/biom10030407 PMC7175247 32155758

[28] R. Vadrevu, C. J. Falzone, C. R. Matthews, Partial NMR assignments and secondary structure mapping of the isolated alpha subunit of Escherichia coli tryptophan synthase, a 29-kD TIM barrel protein. Protein Sci. 12, 185–191 (2003). 12493842 10.1110/ps.0221103 PMC2312393

[29] W. Hu., Stepwise protein folding at near amino acid resolution by hydrogen exchange and mass spectrometry. Proc. Natl. Acad. Sci. U.S.A. 110, 7684–7689 (2013). 23603271 10.1073/pnas.1305887110 PMC3651421

[30] L. E. Rosen, S. V. Kathuria, C. R. Matthews, O. Bilsel, S. Marqusee, Non-native structure appears in microseconds during the folding of E. coli RNase H. J. Mol. Biol. 427, 443–453 (2015). 25311861 10.1016/j.jmb.2014.10.003 PMC4624390

[31] K. Kuwajima, H. Yamaya, S. Sugai, The burst-phase intermediate in the refolding of beta-lactoglobulin studied by stopped-flow circular dichroism and absorption spectroscopy. J. Mol. Biol. 264, 806–822 (1996). 8980687 10.1006/jmbi.1996.0678

[32] K. Kuwajima, H. Yamaya, S. Miwa, S. Sugai, T. Nagamura, Rapid formation of secondary structure framework in protein folding studied by stopped-flow circular dichroism. FEBS Lett. 221, 115–118 (1987). 3040467 10.1016/0014-5793(87)80363-0

[33] K. Kuwata., Structural and kinetic characterization of early folding events in beta-lactoglobulin. Nat. Struct. Biol. 8, 151–155 (2001). 11175905 10.1038/84145

[34] A. Riba., Protein synthesis rates and ribosome occupancies reveal determinants of translation elongation rates. Proc. Natl. Acad. Sci. U.S.A. 116, 15023–15032 (2019). 31292258 10.1073/pnas.1817299116 PMC6660795

[35] R. Zwanzig, A. Szabo, B. Bagchi, Levinthal’s paradox. Proc. Natl. Acad. Sci. U.S.A. 89, 20–22 (1992). 1729690 10.1073/pnas.89.1.20 PMC48166

[36] P. G. Wolynes, J. N. Onuchic, D. Thirumalai, Navigating the folding routes. Science 267, 1619–1620 (1995). 7886447 10.1126/science.7886447

[37] W. Hu, Z.-Y. Kan, L. Mayne, S. W. Englander, Cytochrome c folds through foldon-dependent native-like intermediates in an ordered pathway. Proc. Natl. Acad. Sci. U.S.A. 113, 3809–3814 (2016). 26966231 10.1073/pnas.1522674113 PMC4833275

[38] W. A. Eaton, P. G. Wolynes, Theory, simulations, and experiments show that proteins fold by multiple pathways. Proc. Natl. Acad. Sci. U.S.A. 114, E9759–E9760 (2017). 29087352 10.1073/pnas.1716444114 PMC5699098

[39] S. W. Englander, L. Mayne, The case for defined protein folding pathways. Proc. Natl. Acad. Sci. U.S.A. 114, 8253–8258 (2017). 28630329 10.1073/pnas.1706196114 PMC5547639

[40] R. L. Baldwin, Clash between energy landscape theory and foldon-dependent protein folding. Proc. Natl. Acad. Sci. U.S.A. 114, 8442–8443 (2017). 28747526 10.1073/pnas.1709133114 PMC5559053

[41] S. W. Englander, L. Mayne, The nature of protein folding pathways. Proc. Natl. Acad. Sci. U.S.A. 111, 15873–15880 (2014). 25326421 10.1073/pnas.1411798111 PMC4234557

[42] Y. Wu, R. Vadrevu, X. Yang, C. R. Matthews, Specific structure appears at the N terminus in the sub-millisecond folding intermediate of the alpha subunit of tryptophan synthase, a TIM barrel protein. J. Mol. Biol. 351, 445–452 (2005). 16023136 10.1016/j.jmb.2005.06.006

[43] P. S. Huang., De novo design of a four-fold symmetric TIM-barrel protein with atomic-level accuracy. Nat. Chem. Biol. 12, 29–34 (2016). 26595462 10.1038/nchembio.1966 PMC4684731

[44] P. Löffler, S. Schmitz, E. Hupfeld, R. Sterner, R. Merkl, Rosetta:MSF: A modular framework for multi-state computational protein design. PLOS Comput. Biol. 13, e1005600 (2017). 28604768 10.1371/journal.pcbi.1005600 PMC5484525

[45] B. Höcker, A. Lochner, T. Seitz, J. Claren, R. Sterner, High-resolution crystal structure of an artificial (betaalpha)(8)-barrel protein designed from identical half-barrels. Biochemistry 48, 1145–1147 (2009). 19166324 10.1021/bi802125b

[46] T. Knöchel, A. Pappenberger, J. N. Jansonius, K. Kirschner, The crystal structure of indoleglycerol-phosphate synthase from Thermotoga maritima. Kinetic stabilization by salt bridges. J. Biol. Chem. 277, 8626–8634 (2002). 11741953 10.1074/jbc.M109517200

[47] Z. Y. Kan, X. Ye, J. J. Skinner, L. Mayne, S. W. Englander, ExMS2: An integrated solution for hydrogen-deuterium exchange mass spectrometry data analysis. Anal. Chem. 91, 7474–7481 (2019). 31082210 10.1021/acs.analchem.9b01682

[48] M. Guttman, D. D. Weis, J. R. Engen, K. K. Lee, Analysis of overlapped and noisy hydrogen/deuterium exchange mass spectra. J. Am. Soc. Mass Spectrom. 24, 1906–1912 (2013). 24018862 10.1007/s13361-013-0727-5 PMC3855366

[49] S. El-Gebali., The Pfam protein families database in 2019. Nucleic Acids Res. 47, D427–D432 (2019). 30357350 10.1093/nar/gky995 PMC6324024

[50] B. Schneider., Role of the N-terminal extension of the (betaalpha)8-barrel enzyme indole-3-glycerol phosphate synthase for its fold, stability, and catalytic activity. Biochemistry 44, 16405–16412 (2005). 16342933 10.1021/bi051640n

[51] G. E. Crooks, G. Hon, J. M. Chandonia, S. E. Brenner, WebLogo: A sequence logo generator. Genome Res. 14, 1188–1190 (2004). 15173120 10.1101/gr.849004 PMC419797

[52] A. Drozdetskiy, C. Cole, J. Procter, G. J. Barton, JPred4: A protein secondary structure prediction server. Nucleic Acids Res. 43, W389–W394 (2015). 25883141 10.1093/nar/gkv332 PMC4489285

[53] C. R. Matthews, Effect of point mutations on the folding of globular proteins. Methods Enzymol. 154, 498–511 (1987). 3431461 10.1016/0076-6879(87)54092-7

