# Supplementary material for: A conserved folding nucleus sculpts the free energy landscape of bacterial and archaeal orthologs from a divergent TIM barrel family
Source: Proc Natl Acad Sci U S A. 2021 Apr 19;118(17):e2019571118. doi: 10.1073/pnas.2019571118 (PMC8092565; doi:10.1073/pnas.2019571118)
Supplement: Supplementary File [file pnas.2019571118.sapp.pdf]

PNAS
www.pnas.org

Supplementary Information for

A conserved folding nucleus sculpts the free energy landscape of bacterial and archaeal orthologs from a divergent TIM barrel family.

Rohit Jain[a,1], Khaja Muneeruddin[a,b], Jeremy Anderson[c,2], Michael J. Harms[c], Scott A Shaffer[a,b], C. Robert Matthews[a,3].

*C. Robert Matthews; Department of Biochemistry and Molecular Pharmacology, University of Massachusetts Medical School, 364 Plantation St. LRB 928, Worcester, MA 01605.
Email: C.Robert.Matthews@umassmed.edu

*Rohit Jain; Case Center for Proteomics and Bioinformatics; Case Center for Synchrotron Biosciences, Case Western Reserve University, Cleveland, OH 44106.
Email: rohit.mpibpc@gmail.com

This PDF file includes:

Supplementary text
Figures S1 to S14
Tables S1 to S5
Legends for Dataset S1
SI References

Other supplementary materials for this manuscript include the following:

Dataset S1

Supplementary Information Text

Protein expression and purification

A recombinant version of TmIGPS with the ∆1-31 deletion (to avoid aggregation) and the C101S mutation (to avoid dimerization [4] was employed in our studies. The recombinant TmIGPS was engineered with an N-terminal His6 tag and an intervening TEV Protease site. Expression was done in *E. coli* strain BL21 Codonplus® (DE3)RIL. Ultrapure urea, deionized using AG® 501-X8 (D) Resin (Bio-Rad), was used for His6-TmIGPS purification under unfolding conditions (7.2 M urea). Bacterial cells were lysed in 50 mM Tris-HCl, 10 mM KCl, 0.8% Triton® X-100, 2 M urea at pH 7.2 and using a French press. The inclusion bodies were pelleted and then solubilized overnight in 8 M Urea buffer without detergent. The soluble fraction after centrifugation was bound to Ni-NTA resin for 8 hours at 4°C. The bound fraction on Ni-NTA resin was thoroughly washed, and the His6-tagged TmIGPS fraction was eluted with a step gradient of 10 mM, 20 mM and 400 mM imidazole in 8 M Urea buffer. The pure fraction in 400 mM imidazole was dialyzed into 8 M Urea buffer to remove imidazole.

A drop-wise dilution procedure was used for refolding protein in 8 M Urea buffer into 0.9 M Urea buffer. His6-tagged TEV protease was used to cleave the His6-tag from His6-TmIGPS in overnight incubation at 4°C. The cleavage product was applied to a DEAE-Sepharose column and eluted using a salt gradient of 0 M to 1 M KCl in 10 mM KPi buffer at pH 7.2. Fractions with His6-tag cleaved TmIGPS were pooled and incubated in Ni-NTA resin for 1 hour. The unbound fraction of TmIGPS (without His6 tag) was collected after elution. The protein was concentrated and then purified by a final size-exclusion purification step on a Superdex-75 gel filtration column. TmIGPS purity was confirmed (> 98%) with SDS PAGE and ESI-MS measurement on a Waters Q-TOF ESI mass spectrometer. The concentrations of the purified TmIGPS (without His6-tag) were determined from the UV absorbance at 280 nm and the corrected (1) extinction coefficient of 18428 M^{-1} cm^{-1}.

Equilibrium unfolding and refolding experiments with CD and FL spectroscopy

The far-UV CD and tryptophan fluorescence emission spectra were collected in equilibrium experiments to determine TmIGPS stability. Far-UV CD spectroscopy was employed to monitor the secondary structure on a Jasco Model J-810 spectropolarimeter. CD data were collected from 200 nm to 260 nm using a quartz cuvette of 5 mm pathlength. CD data were obtained at a scan rate of 50 nm/min, with intervals of 1 nm, a bandwidth of 2.5 nm and an averaging time of 8 s. Three replicate spectra were collected and averaged. Tryptophan fluorescence was collected on a T-format Horiba Fluorolog fluorimeter. The emission spectra from excitation at 280 nm were collected between 300 and 450 nm at a 1 nm interval and averaged over three traces. CD and fluorescence measurements were done at 25 °C and the temperature was maintained with water-cooled Peltier temperature control systems.

A Hamilton MICROLAB 5000 automatic titrator was used to prepare the samples for equilibrium unfolding and refolding experiments. Samples for the unfolding titrations were prepared by 1:10 mixing of appropriate volumes of 0 M and 8 M GdnHCl buffers to a 28 µM stock of native TmIGPS in buffer. The final concentration of TmIGPS in unfolding titrations was 2.8 µM. Samples for the refolding titrations were prepared by 1:30 mixing of appropriate volumes of 0 M and 8 M GdnHCl buffers to a 98.4 µM stock of unfolded TmIGPS in 7.2 M GdnHCl. The final concentration of TmIGPS in refolding titrations was 3.3 µM. The buffer contained 10 mM KPi (pH 7.2) and 10 mM KCl. The GdnHCl concentration increment was 0.2 M to enhance the precision of the measurements.

Samples were incubated at 25 °C for up to 9 days. Aliquots were periodically withdrawn for CD and fluorescence measurements after 1 day / 6 days / 9 days. The progress of the unfolding and refolding reactions was monitored until 9 days when the titration cuves were coincident, i.e., at equilibrium. The GdnHCl concentration for all samples was determined by measuring the refractive index on a Leica Mark II refractometer (2). The far-UV CD data for unfolding and refolding after 9 days were fit to a three-state model using an in-house algorithm, Savuka version 6.2 (3). The

change in tryptophan emission at 352 nm as a function of GdnHCl concentrations was fit to two-state model with Savuka version 6.2.

Kinetic refolding and unfolding experiments with CD and FL spectroscopy

Manual mixing was used to initiate the slow unfolding and refolding reactions, > 10 s, for TmIGPS. The change in ellipticity as a function of time was monitored on a Jasco Model J-810 spectropolarimeter. CD data were collected at 222 nm in a quartz cuvette of 5 mm pathlength. The change in tryptophan emission (excitation = 280 nm, emission = 352 nm) as a function of time in unfolding and refolding reactions was collected on a T-format Fluorolog fluorimeter. CD and fluorescence measurements were done at 25 °C and the temperature was maintained with water-cooled Peltier temperature control systems.

The dead-time of the manual mixing experiments was 3 s, and the instrument response time was about 5 s. Unfolding experiments with manual mixing were initiated from the native state in 10 mM KPi (pH 7.2) and 10 mM KCl to final GdnHCl concentrations ranging from 1.25 M to 4.5 M. Refolding experiments with manual mixing were initiated from the unfolded state at 7.2 M GdnHCl to final GdnHCl concentrations ranging from 1.1 M to 0.2 M. The final solution volume was 2 ml, and final GdnHCl concentrations were measured by Leica Mark II refractometer.

The faster unfolding and refolding kinetics measurements were monitored with stopped-flow instruments. CD data were collected at 222 nm using an AVIV-202 stopped-flow spectrophotometer with a dead time of 5 ms. Measurements were done at 25°C with water-cooled Peltier temperature control system. Stopped-flow fluorescence experiments were performed on an Applied Photophysics instrument (SX.18MV) with a dead-time of 2 ms. The excitation wavelength was 280 nm, and the emission was monitored using a 320 nm cutoff filter. The path lengths for excitation and emission were 10 mm and 2 mm, respectively. A thermostatically regulated water bath was used to maintain the flow-cell temperature at 25° C during fluorescence measurements.

Unfolding experiments with stopped-flow CD measurements were performed by jumping from 0 M to different GdnHCl concentrations (3 M – 6 M). Unfolding experiments with stopped-flow fluorescence measurements were performed by jumping from 0 M to different GdnHCl concentrations (2 M – 6.8 M). Refolding experiments with stopped-flow fluorescence were initiated by jumping from the unfolded state at 7.2 M GdnHCl to final GdnHCl concentrations ranging from 1.5 M to 0.5 M. The kinetic unfolding and refolding data at different GdnHCl concentrations were fitted to a series of exponential functions using a nonlinear least-squares fitting with OriginPro and Savuka (3, 4). The exponential fitting gave relaxation times and amplitudes associated with kinetic experiments. The $\log_{10}$ values of the relaxation times were plotted as a function of the final GdnHCl concentration.

Hydrogen/Deuterium exchange experiments for intact protein with mass spectrometry.

Deuterium uptake in intact TmIGPS was determined by a home-built HDX module and UPLC ESI-MS. Chromatographic separations were performed using a Waters Acquity UPLC instrument and a C4 reverse-phase column (Waters Protein BEH 300Å, 1.7 µm, 2.1 mm × 50 mm). A LC method of 2 min wash (25% acetonitrile) followed by a 7 min v/v gradient (25% - 75% acetonitrile, 0.2% formic acid) was used for elution at a flow-rate of 250 µl/min. Each LC run for a protein sample was followed by 3 blank runs (50% isopropanol) with gradient (25% - 75% acetonitrile, 0.2% formic acid) to minimize carry over. Temperature inside HDX module was maintained at 0 °C with dry ice to minimize back exchange. Temperature was monitored using a temperature probe. The eluent was transferred in a water-cooled tube to a Synapt G2-Si ESI mass spectrometer (Waters) operating in the positive ion electrospray mode for ionization and detection. A LockMass solution with the Glu-fib peptide (*m/z* 785.8426) was run during ESI-MS measurements for offline mass correction.
The H-to-D exchange behavior of 50 µM TmIGPS was monitored at different GdnHCl concentrations after equilibration for 9 days. The final GdnHCl concentration for all samples was determined by measuring the refractive index on a Leica Mark II refractometer. Equilibration at

various GdnHCl concentrations was followed by a 1:20 pulse of same deuterated D_2O/GdnHCl concentration for 10 s at pD 7.2 (pH meter reading = 6.8) and 25 °C. The sample was then diluted 1:5 on ice with cooled with 200 mM potassium phosphate to reduce the pH to 2.5. A small volume of ice-cold protonated 7 M GdnHCl at pH 2.5 (0.2% formic acid) was added in quenched samples so that all samples had ~1 M GdnHCl before loading on HDX module. 50 µl quenched samples containing ~620 ng intact TmIGPS were injected manually and using a cooled glass syringe on a home built HDX module. The H-to-D exchange rates for TmIGPS backbone NHs at pD 7.2 and 25 °C were predicted by the SPHERE program (5).

The average time constant is 610 ms for all exchangeable backbone amides in intact TmIGPS. The ESI-MS measurement for 0% reference represents the unfolded state of TmIGPS that has not been exposed to D_2O, yielded the molecular mass expected for the sequence of the intact protein (observed, 24,859.4 Da; theoretical, 24859.6 Da). The number of exchangeable backbone NHs in TmIGPS is 212 (Total residues, 222; Prolines, 9). Experimental conditions were kept at pH 2.5 and 0 °C for quenching and chromatographic steps to minimize back exchange (D-to-H) rates. The 100% reference measurement had an uptake of 181 deuteriums and represents the unfolded state that has been exposed overnight to deuterated 6 M D_2O/GdnHCl. The uptake of 181 deuterium out of total 212 exchangeable backbone NHs corresponds to a back exchange percentage of 15 % in our experimental setup.

The ESI-MS spectrum for every measurement was mass corrected with a LockMass measurement and Waters MassLynx software. Gaussian peak fitting was used to analyze the +28 charge state mass spectrum at different experimental conditions with softwares OriginPro and Savuka 6.2. The number of exchanged backbone amides and relative peak area obtained after Gaussian fitting were plotted from 0M – 6M GdnHCl concentrations. Reference measurements (0% and 100% controls) were done for measurements on different days.

Hydrogen/Deuterium exchange experiments with online pepsin proteolysis and mass spectrometry.

Deuterium uptake at the peptide level was determined with a home-built HDX module and UPLC ESI-MS. A proteolytic pepsin column (Waters Enzymate BEH, 300 Å, 5 µm, 2.1 × 30 mm) was connected online to a sample loop in the HDX module. A Thermo pump was used to continuously run 0.2 % formic acid (pH 2.5) through the pepsin column at 100 µl/min under isocratic conditions. TmIGPS was digested and the resulting peptides were trapped on a C18 reverse phase VanGuard Pre-column (Waters Peptide BEH, 300 Å, 1.7 µm, 2.1 mm × 5 mm). Peptides separations were performed using a Waters Acquity UPLC instrument and a C18 reverse-phase column (Waters Peptide BEH 300 Å, 1.7 µm, 1 mm × 100 mm). A LockMass solution with the Glu-fib peptide (*m/z* 785.8426) was run during ESI-MS for offline mass correction.

The H-to-D exchange behavior of 40 µM TmIGPS was monitored at different GdnHCl concentrations after equilibration for 9 days. The equilibration was followed by 1:12 pulse of same deuterated D_2O/GdnHCl concentration for 10 s at pD 7.2 (pH meter reading = 6.8) and 25 °C. The sample was then diluted 1:5 on ice with cooled with 200 mM potassium phosphate to reduce the pH to 2.5. A small volume of ice-cold protonated 7 M GdnHCl at pH 2.5 (0.2% formic acid) was added in quenched samples so that all samples had ~1 M GdnHCl before loading on HDX module. 50 µl quenched samples containing ~800 ng intact TmIGPS protein were loaded with a cooled glass syringe on a sample loop in the HDX module. The “Load" position was used to fragment the protein and capture peptides on the trap column for desalting in a 3 min run with 0.2% formic acid at 100 µl/min. The valve was manually switched after 3 min to the “Inject” position. In this position, a 12 min v/v gradient (5% - 60% acetonitrile, 0.2% formic acid, 50 µl/min) flow was used to elute the peptides from the trap column (BEH C18, Waters) and separate them on the BEH C18 (Waters) column. The eluent after peptide separation was transferred in a water-cooled tube to a Synapt G2-Si ESI (Waters) mass spectrometer operating in the positive ion electrospray mode for ionization and detection. Each LC run for a protein sample was followed by 3 blank runs (50% isopropanol) with gradient (5% - 60% acetonitrile, 0.2% formic acid) to minimize carry over. Temperature in the

HDX module was maintained at 0°C with dry ice to minimize back exchange of deuterium during proteolysis and peptide separation steps. Temperature was monitored using a temperature probe. The all-hydrogen/undeuterated TmIGPS sample (0 % reference) was run initially to determine the retention time (RT) of each peptide. The all-deuterium/fully deuterated TmIGPS sample (100 % reference) was then run to calibrate the recovery of the deuterium-label for each peptide. The reference measurements were followed by deuterated samples for different experimental conditions. Reference measurements (0% and 100% controls) were done for measurements on different days.

The generation of the peptide list in MS/MS scans of the all-hydrogen/undeuterated sample was automated while the search and validation of peptides in HDX-MS experiments was semi-automated. The ProteinLynx Global Server (PLGS, WATERS) was used to identify and analyze the MS/MS scan of proteolytically digested peptides in all-hydrogen/undeuterated TmIGPS sample. The ESI-MS spectrum for every measurement was mass corrected with a LockMass measurement using Walers MassLynx software. The ExMS2 program was then used to search and validate peptides listed in PLGS output through MS scan of all-hydrogen/undeuterated sample (6). The mass spectra of peptides were compared with their natural isotopic distribution and local spectral features. This procedure accurately established the chromatographic retention time (RT range) of peptides that passed various tests in ExMS2 search. The RT range of passed peptides in all-hydrogen/undeuterated sample was used to search and validate peptides in MS scans of experimental deuterated samples. A manual check was done to accept or reject assignments for all peptides in different experimental conditions. The ambiguous results were corrected by RT range adjustment and manual cleaning.

Peptides in experimental conditions presented both EX1 and EX2 hydrogen exchange behavior. The spectral envelope of every peptide was converted to a centroid distribution and one or more populations were fitted with binomial functions at each GdnHCl concentration. The fitting results were manually checked for all peptides and were corrected when required. The hydrogen exchange profile of each peptide with its population and deuterium uptake at increasing GdnHCl concentration was plotted for comparison. After binomial fitting, the centroid mass of each peptide from all-H, all-D and partially-deuterated samples was calculated with ExMS2. The centroid mass was used to calculate and plot relative deuterium uptake by each peptide in different experimental conditions with OriginPro.

Determination of BASiC hydrophobic clusters

BASiC Networks is a lab-based program that performs cluster analysis of hydrophobic contacts in protein structures, based on the “Contacts of Structural Units” (CSU) algorithm [9, 10]. The program was used to calculate the surface area between atoms in TmIGPS (pdb 1I4N) and SsIGPS (pdb 2C3Z) crystal structures (7, 8). The contacts between side-chain carbon atoms of the residues with **B**ranched **A**liphatic **Si**de **C**hains (BASiC), isoleucine, leucine and valine (ILV), were selected for analysis (9, 10). A BASiC network is comprised of pair-wise ILV contacts having > 10 Å^2 surface area burial.

SI Figures

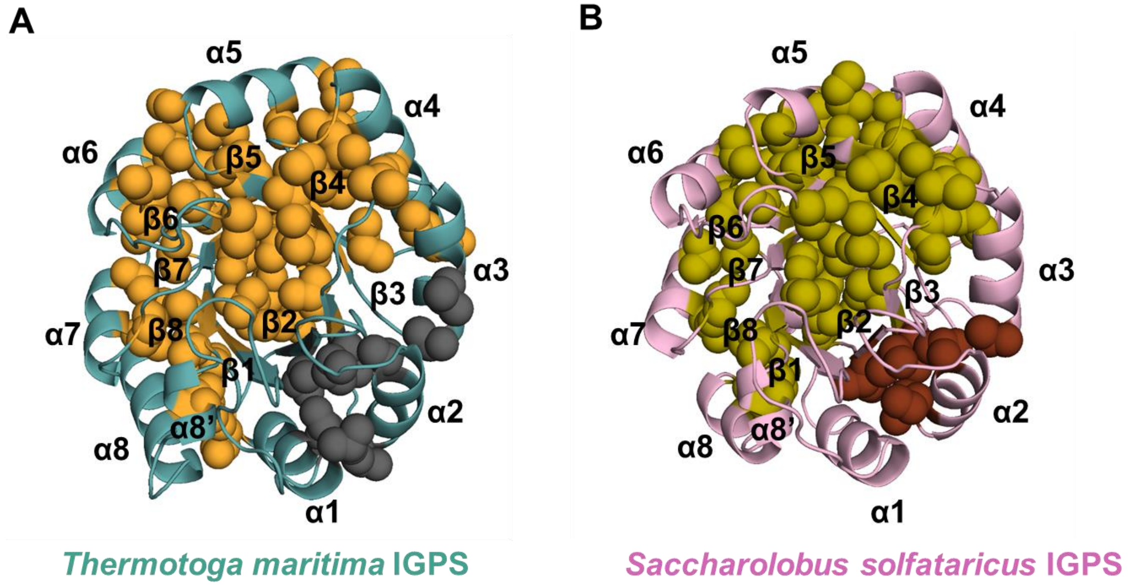

Fig. S1. ILV clusters in (A) TmIGPS (PDB:1I4N) and (B) SsIGPS (PDB:2C3Z). The aliphatic side chains from ILV (PDB:1I4N) residues were overlaid as spheres on the ribbon diagram of two IGPS orthologs. Clusters are defined as the set of residues whose pairwise contact surface area is or exceeds 10 Å^2 (9).

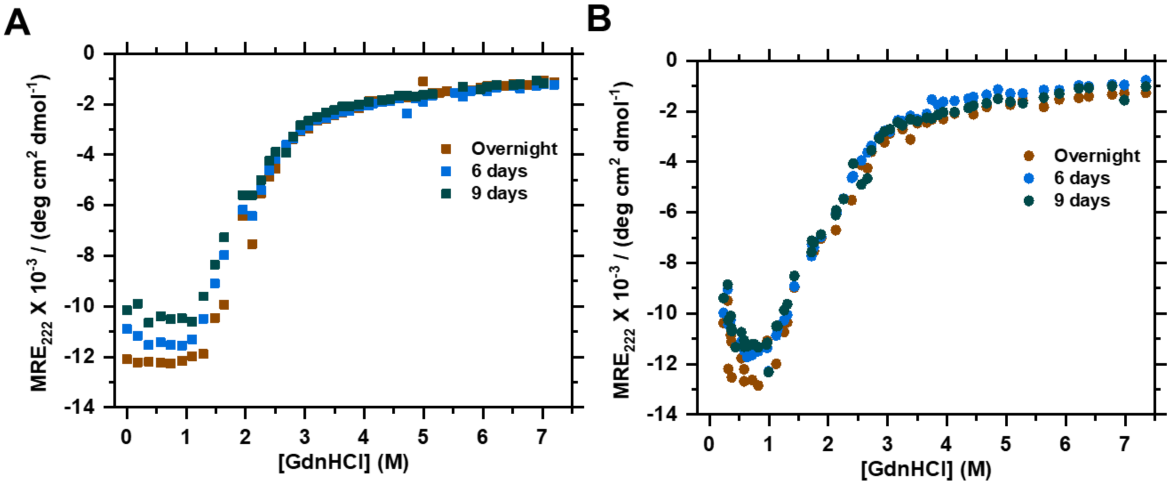

Fig. S2. (A) Unfolding and (B) refolding titrations of TmIGPS with GdnHCl monitored by far-UV CD spectroscopy; 9 days are required to reach equilibrium at pH 7.2 and 25° C. The mean residue ellipticity at 222 nm was plotted against final GdnHCl concentration. The buffer in all experiments contained 10 mM potassium phosphate and 10 mM KCl. The increase in the ellipticity between 0 M and 1.5 M GdnHCl with time reflects the loss of signal due to aggregation.

Table S1: Thermodynamic and kinetic parameters for the folding reactions in TmIGPS. The CD equilibrium data were fit to a 3-state model, assuming a linear dependence of the free energy of the folding reactions on the denaturant concentration (11). The *m*-value represents the linear dependence on denaturant concentration and the Z parameter represents the fractional loss of ellipticity at 222 nm in the I_{eq} intermediate. The FL data were fit to a 2-state model with the same assumptions.

Experiment	EQ unfolding with CD		EQ unfolding with FL
Folding reaction	$N \rightleftharpoons I_{eq}$	$I_{eq} \rightleftharpoons U$	$N \rightleftharpoons I_{eq}$
$\Delta G°$ / kcal mol^{-1}	5.4 ± 0.1	4.1 ± 0.2	4.9 ± 0.6
m-value / kcal mol^{-1} (M $urea)^{-1}$	3.7 ± 0.8	1.7 ± 0.1	3.3 ± 0.4
Z parameter	0.55 ± 0.1		-

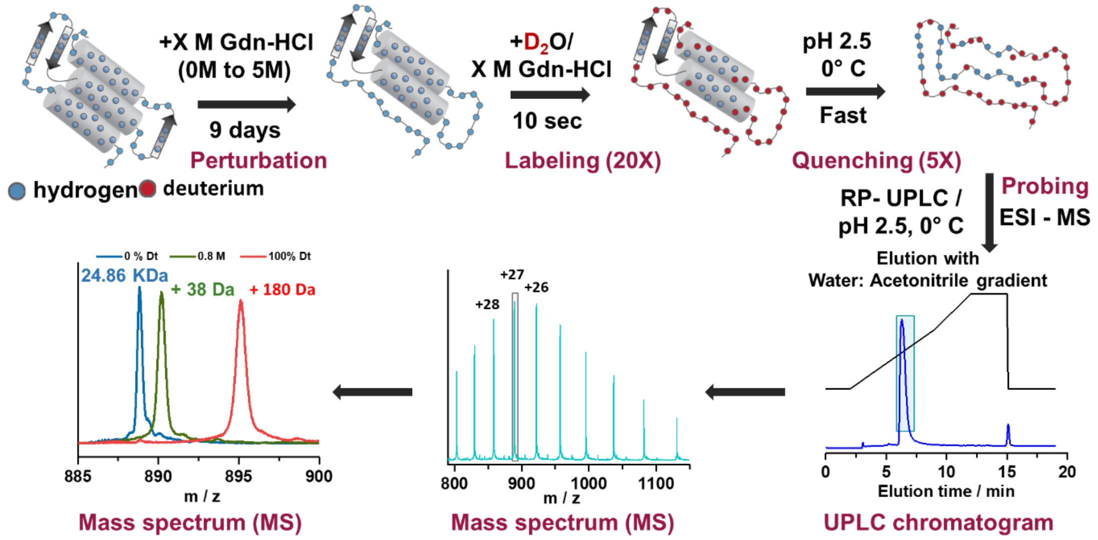

Fig. S3. The schematic for the equilibrium HDX-MS experiments at intact level which were used to study the TmIGPS folding. Please refer to Methods and Supplementary text sections for more details.

Table S2: The deuterium uptake and the relative peak area of +28 charge state for different TmIGPS transitions in intact equilibrium HDX-MS experiments shown at selected GdnHCl concentrations. Dt designates the maximum observed deuterium uptake; the expected maximum uptake is 213 Da. The difference reflects the 15 % back-exchange during the work flow.

Folding reaction	$N \rightleftharpoons N'$		$I_{bp} \rightleftharpoons U$		$I_a \rightleftharpoons U$	
GdnHCl / M	Deuterium uptake / Da	Rel. peak area	Deuterium uptake / Da	Rel. peak area	Deuterium uptake / Da	Rel. peak area
0	34 ± 0.1	100	-	-	-	-
0.3	38 ± 0.1	100	-	-	-	-
0.8	43 ± 0.5	100	-	-	-	-
1.1	46 ± 0.5	100	-	-	-	-
1.3	44 ± 0.1	45	109 ± 2.2	33	132 ± 0.6	23
1.8	-	-	135 ± 0.1	73	147 ± 0.1	27
2.5	-	-	167 ± 1.6	50	172 ± 0.1	50
2.8	-	-	-	-	167 ± 0.1	100
6	-	-	-	-	180 ± 0.1	100
100% Dt	-	-	-	-	181 ± 0.1	100

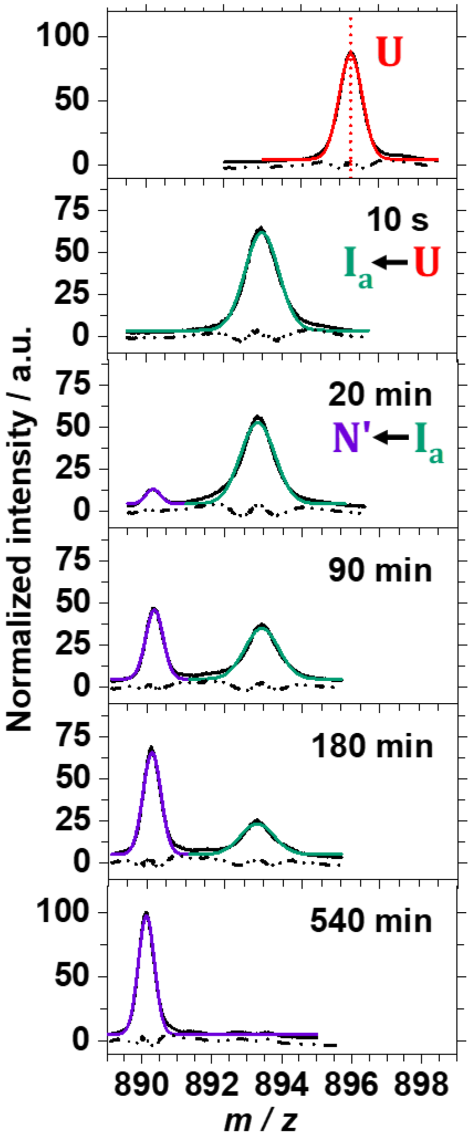

Fig. S4. The ESI-MS spectra for the +28 charge state at specified times during the refolding of TmIGPS from 6.2 to 0.8 M GdnHCl. The spectra were fit with Gaussian functions and assigned to species on the refolding pathway, according to the kinetic mechanism. The refolding TmIGPS was pulse labeled at different time points with 0.8 M D_2O/GdnHCl for 10 s, quenched and monitored with HDX-MS. Normalized ESI-MS spectra (solid black curves) were fitted with Gaussian functions. Residual curves are shown (dash-dot-dot black lines).

Table S3: Intact equilibrium (EQ) and kinetic (KN) HDX-MS experiments are compared by their deuterium uptake and gaussian peak width for +28 charge state at selected GdnHCl concentrations. In the kinetic experiments, TmIGPS was refolded from 6.2 M GdnHCl to the final concentration indicated in the table. After refolding for 10 s, samples were pulse labeled with the same final D_2O/GdnHCl concentration for 10 s, quenched and monitored with HDX-MS. The higher deuterium uptake of the I_a state in KN experiments after refolding for 10 s is likely due to the incomplete equilibration of I_a and I_{bp}.

State	EQ - N′		EQ - I_{bp}		EQ - I_a		KN - I_a	
GdnHCl / M	Dt. uptake / Da	Width	Dt. uptake / Da	Width	Dt. uptake / Da	Width	Dt. uptake / Da	Width
0.6*	42 ± 0.1	0.23 ± 0.01	-	-	-	-	116 ± 0.1	0.43 ± 0.01
0.8*	46 ± 0.5	0.23 ± 0.01	-	-	-	-	116 ± 0.1	0.43 ± 0.01
1.3	43 ± 0.1	0.25 ± 0.01	109 ± 2.2	0.89 ± 0.1	132 ± 0.6	0.42 ± 0.02	147 ± 0.1	0.42 ± 0.01
1.8	-	-	135 ± 0.1	0.72 ± 0.01	147 ± 0.1	0.30 ± 0.01	162 ± 0.1	0.35 ± 0.01

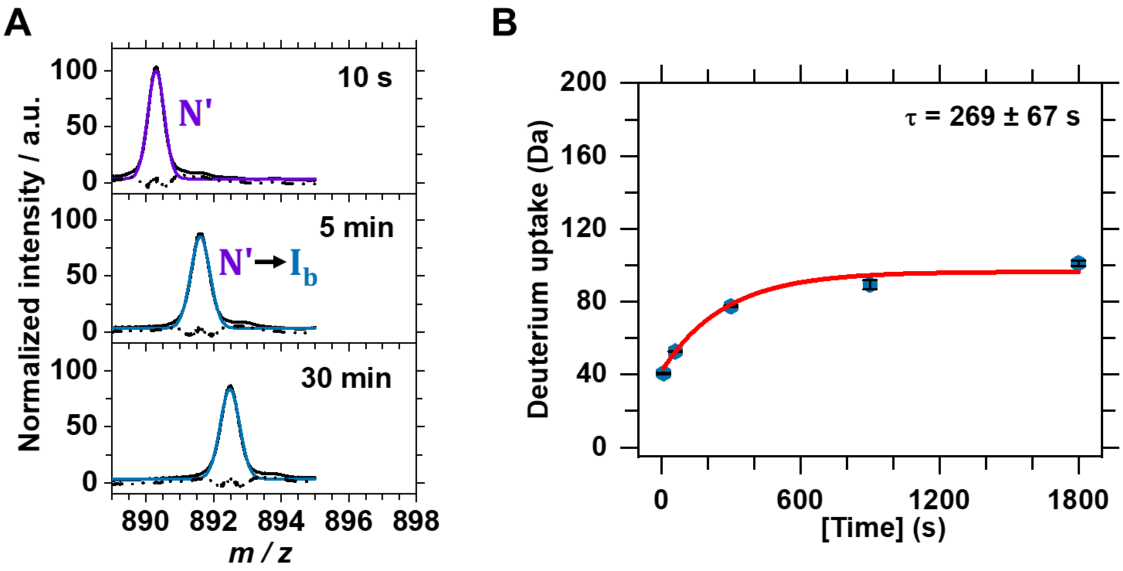

Fig. S5. H-to-D exchange for the N′ to I_b transition. (A) Exchange from the N' state at 1.5 M GdnHCl through the intermediate, I_b (blue) state was followed by the spectrum of the +28 charge state as a function of time. (B) The deuterium uptake was fitted to a single exponential function (τ = 269 ± 67 s, red line).

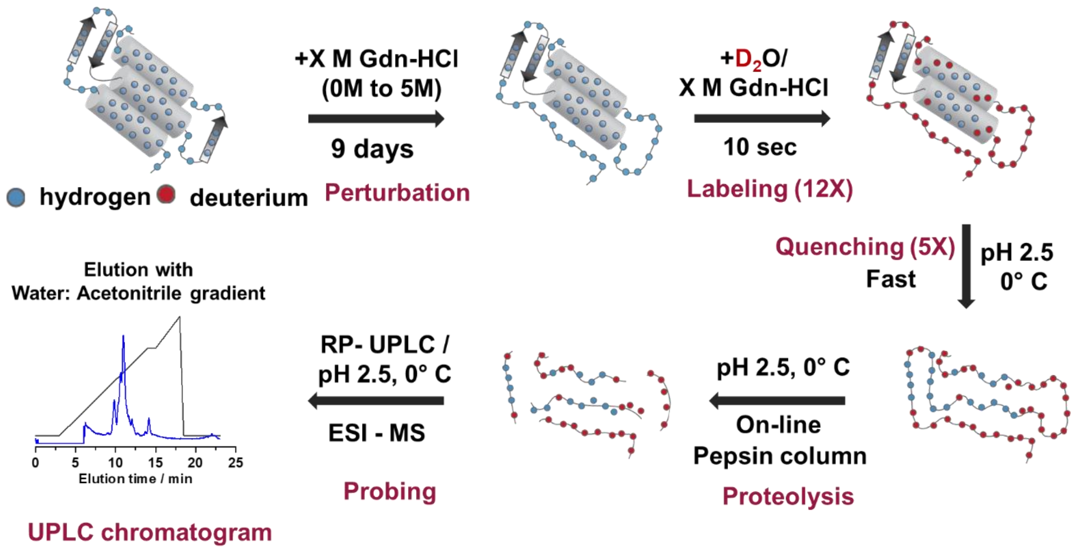

Fig. S6. The schematic for the equilibrium HDX-MS experiments at peptide level which were used to study the TmIGPS folding. Please refer to Methods and Supplementary text sections for more details.

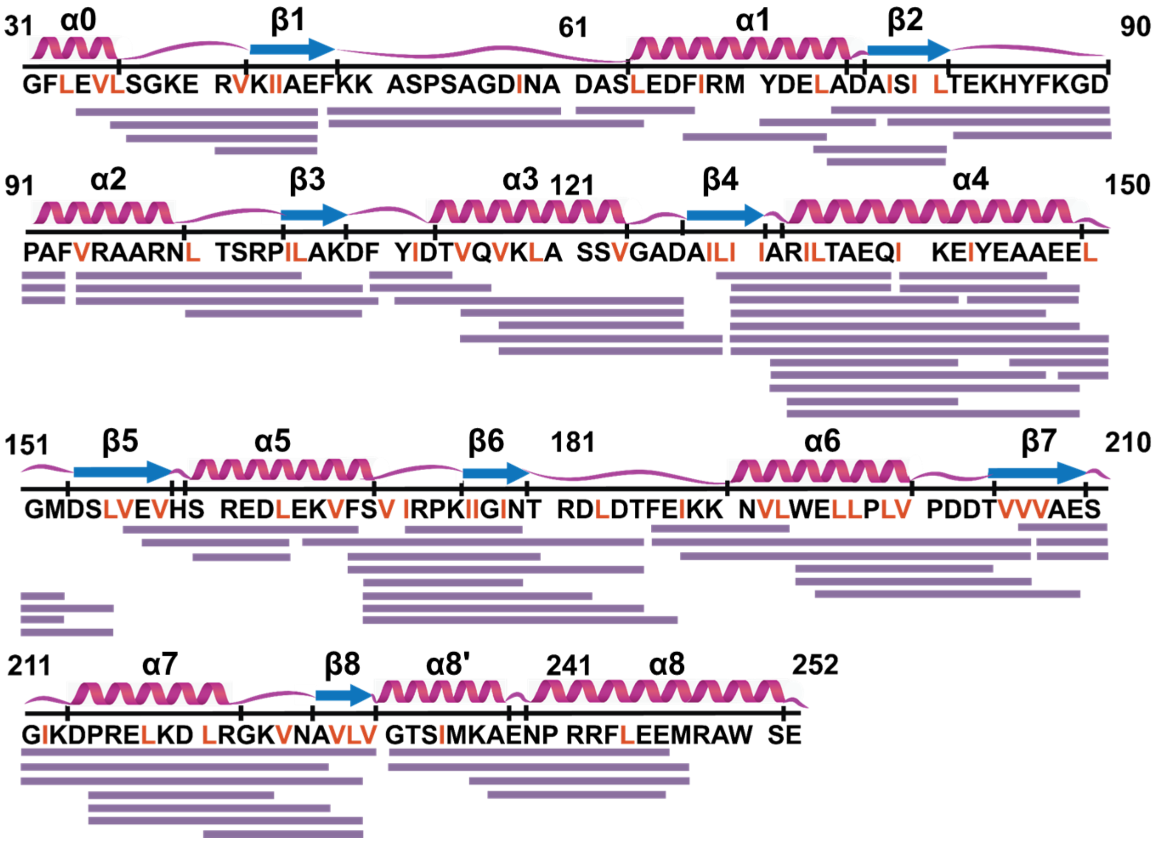

Fig. S7. The peptide map of TmIGPS after online pepsin proteolysis in HDX-MS experiments. 70 peptides that cover 97% of the amino acid sequence in TmIGPS were used for analyzing the protection patterns of various species on the TmIGPS folding free energy surface. Secondary structure elements are labeled and displayed on the top of amino acid sequence. The α-helices are shown in magenta, β-strands in blue, and the peptides are displayed in lavender. Isoleucine, leucine and valine residues are colored red.

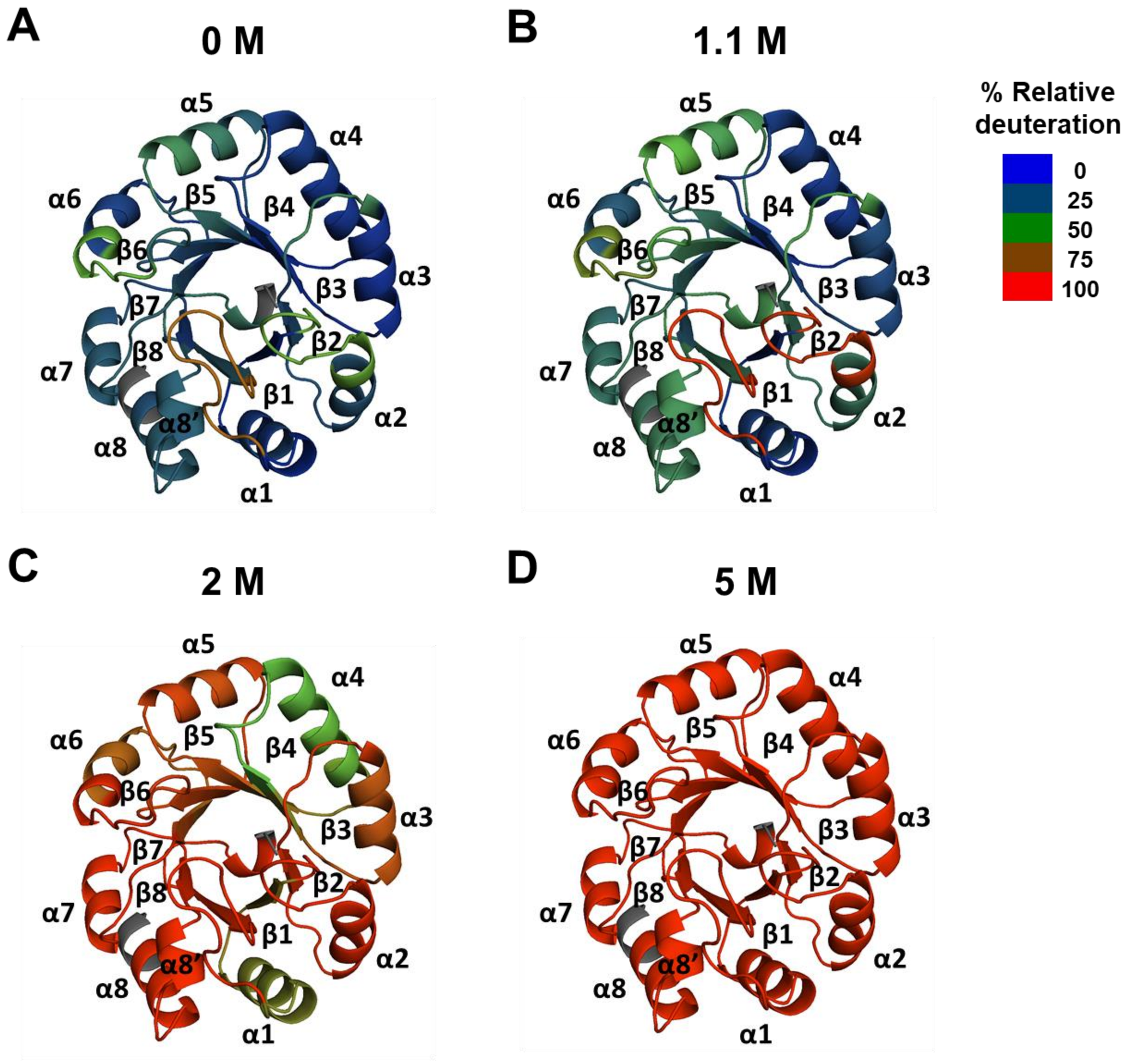

Fig. S8. The relative deuteration (%) of TmIGPS segments for highly populated species on the folding free energy surface from equilibrium HDX-MS experiments. (A) N state at 0 M GdnHCl. (B) N′ state at 1.1 M GdnHCl. (C) I_a and/or I_{bp} states at 2 M GdnHCl. (D) U state at 5 M GdnHCl. Segments not covered by pepsin proteolysis are colored gray.

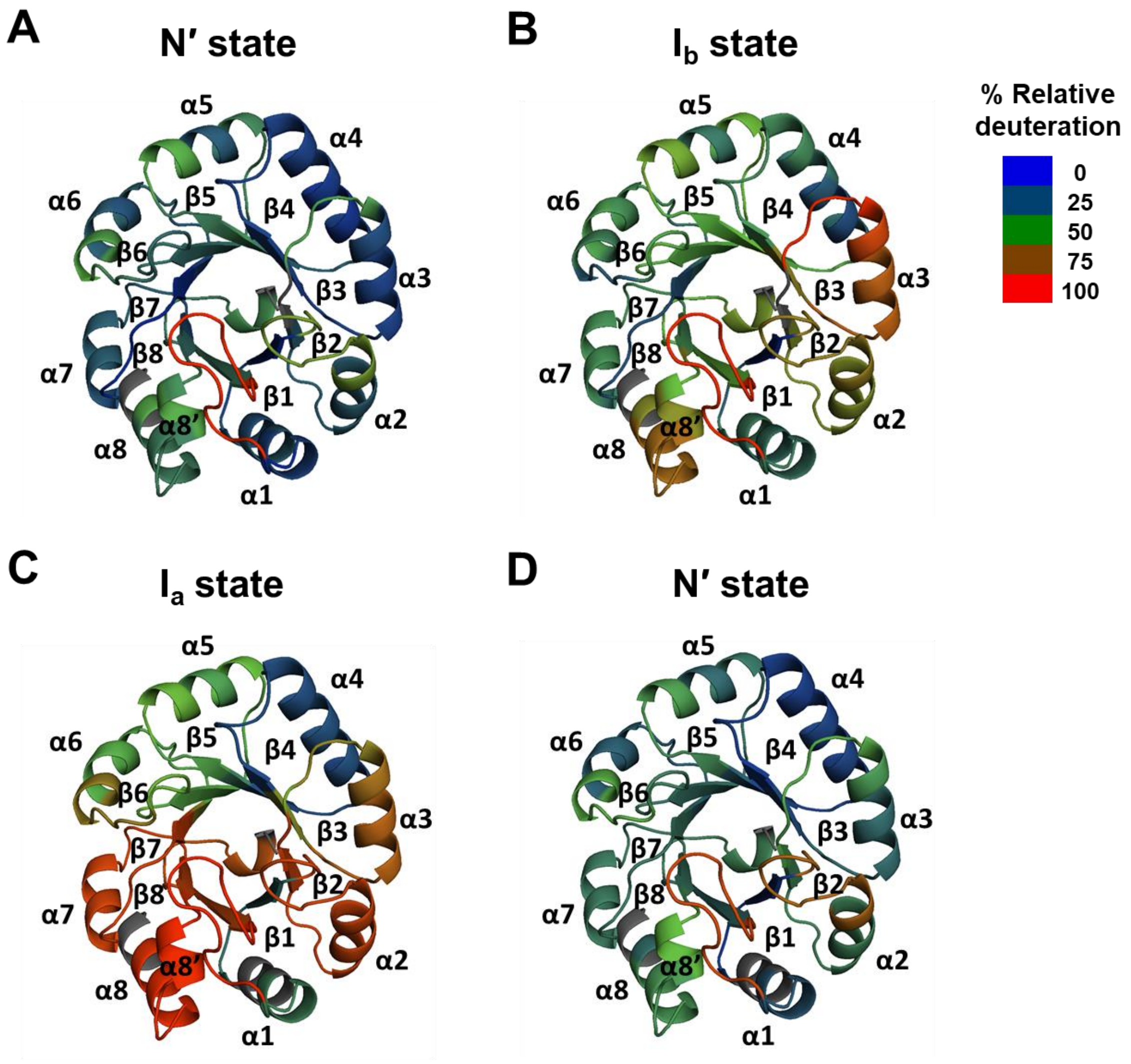

Fig. S9. The relative deuteration (%) of TmIGPS segments for highly populated species on the folding free energy surface from kinetic HDX-MS experiments. The unfolding was initiated by incubating native TmIGPS in deuterated 1.5 M D_2O/GdnHCl. The (A) N′, 10 s and (B) I_b, 30 min samples were labeled for 10 s, protelolyzed and analyzed with HDX-MS. The deuterium labeling time was determined from intact HDX-MS experiments (SI Appendix, Fig. S5). In a separate experiment, refolding was initiated by diluting unfolded TmIGPS (6.2 M GdnHCl) to native conditions (0.8 M GdnHCl). The refolded samples (C) I_a, 10 s N′ and (D) N′, 570 min were deuterium labeled with 0.8 M D_2O/GdnHCl for 10 s, proteolyzed and analyzed with HDX-MS. The refolding time was determined from intact HDX-MS experiments (SI Appendix, Fig. S4). Segments not covered by pepsin proteolysis are colored gray.

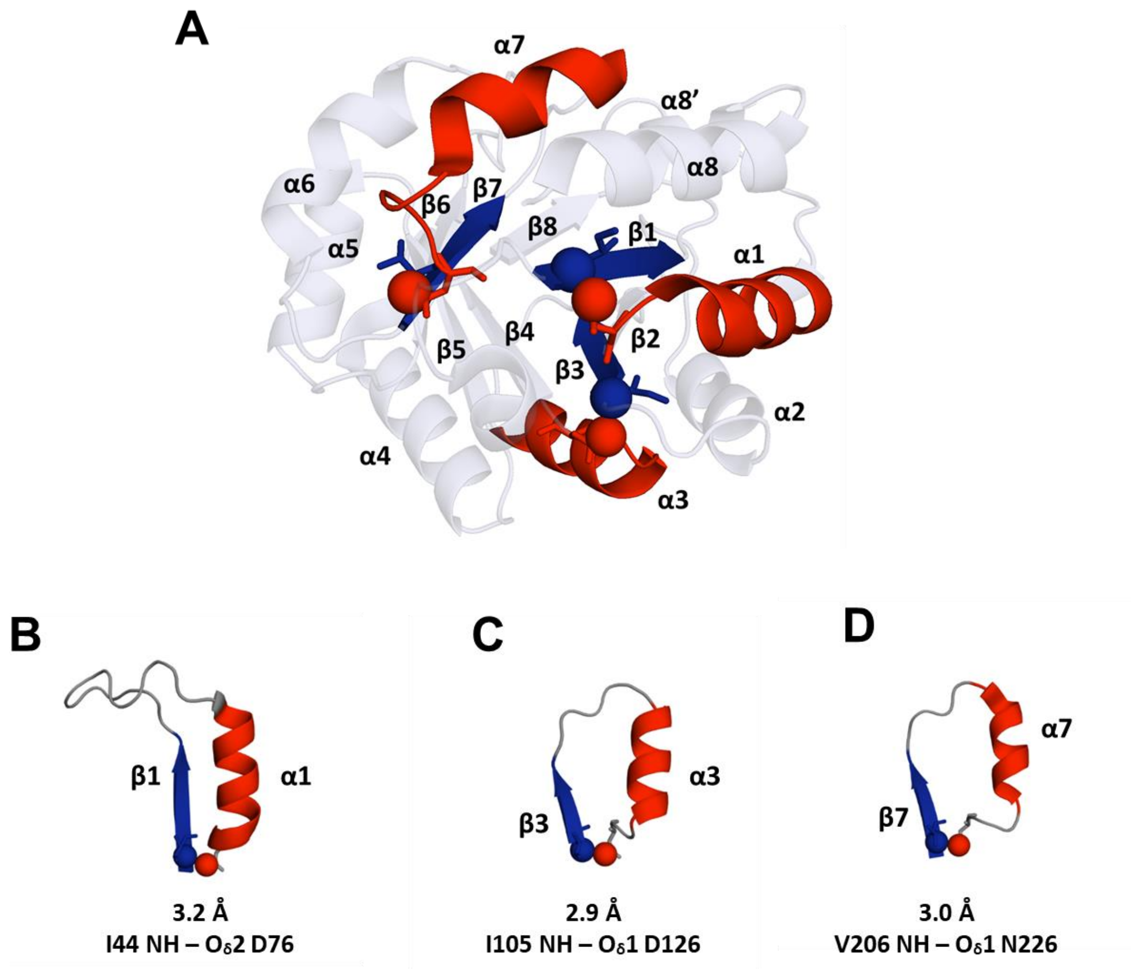

Fig. S10. Ribbon diagram (A) highlighting the βα-hairpin clamps in TmIGPS. βα-hairpin clamps are the non-local hydrogen bonding interactions between main chain amide hydrogen atoms and polar side chain acceptors that bracket the consecutive βα or αβ secondary structure elements in TIM barrels (12). Residues forming clamps in TmIGPS are displayed along with their intervening secondary structures elements (B, C and D). The H-bond donor (blue) and acceptor (red) atoms are colored and the distances between the donor and acceptor atoms are indicated.

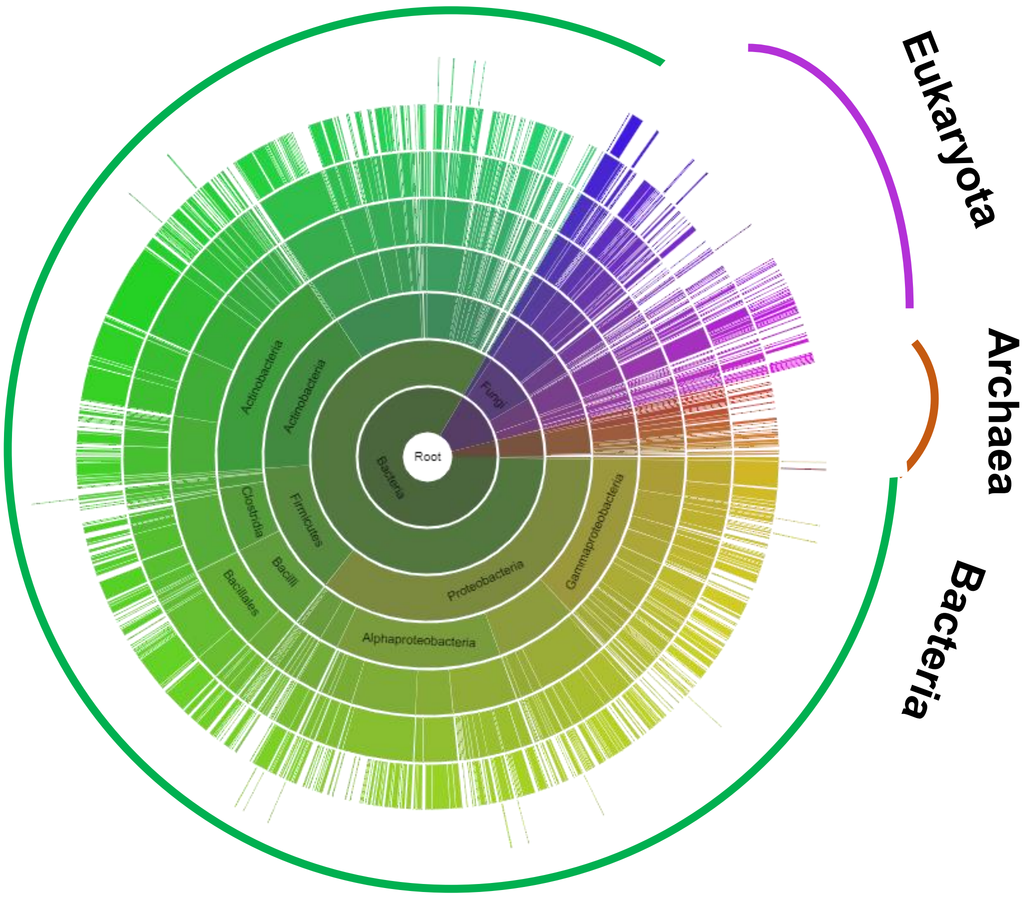

Fig. S11. Sunburst visualization of the evolution tree for the IGPS family of TIM barrels across all kingdoms. Segments of tree are colored according to their superkingdom - Bacteria (green), Archaea (orange) and Eukarya (purple). Different arcs in the sunburst tree represent nodes in the taxonomic lineage of IGPS sequences. The taxonomic level is depicted by the radius of the arc and its distance from the root node at the center of the sunburst. The length of the arc represents either the number of IGPS sequences at that taxonomic level, or the number of species that are found beneath the node in the tree. This figure was taken from Pfam database (13) hosted by EMBL-EBI (https://pfam.xfam.org/family/IGPS#tabview=tab0).

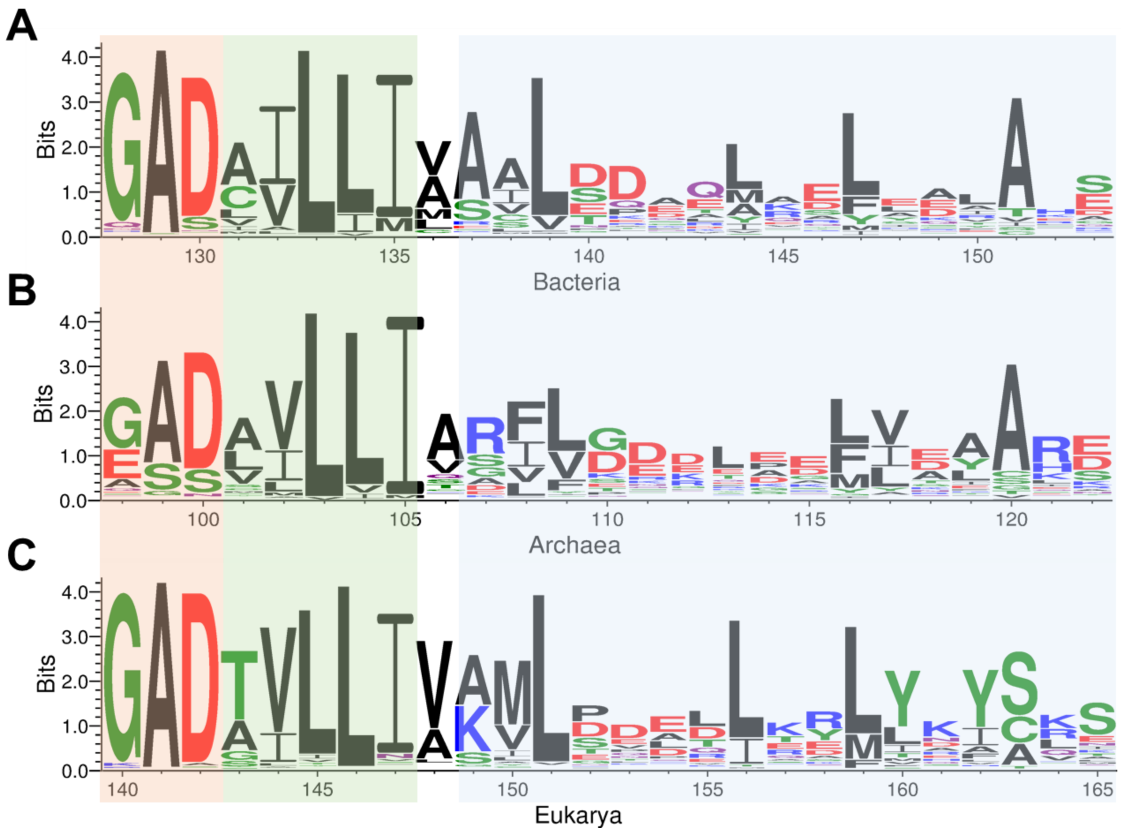

Fig. S12. Sequence logos from aligned IGPS amino acid sequences show the conservation of aliphatic amino acids (black) in β4 (green) and α4 (blue) segments from (A) Bacteria, (B) Archaea and (C) Eukarya. Adjacent leucine-leucine-isoleucine residues in β4 strand are strongly conserved for all 3 superkingdoms. The β4α4 segment is preceded by the highly conserved hairpin clamp (GAD, orange).

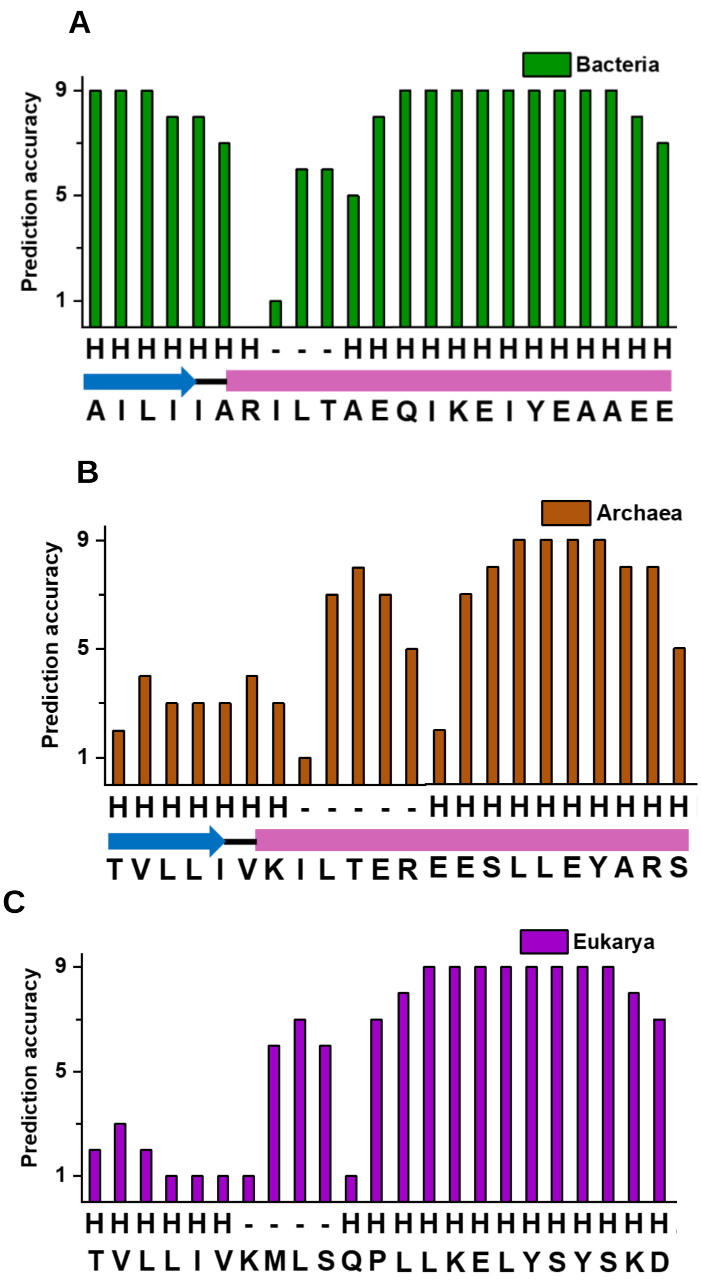

Fig. S13. Predicted secondary structure for β4α4 segment in the IGPS reference sequence. (A) Bacteria (*T. maritima*), (B) Archaea (*S. solfataricus*) and (C) Eukarya (*S. cervisiae*). The multiple sequence alignment (< 95 % identity) for each kingdom was used for secondary structure prediction with JPRED4. The predicted secondary structure is marked as H (helix) and – (coiled coil) along with prediction accuracy (0 – 9). The sequence of the β4α4 segment for the reference sequence is shown along with its secondary structure from the crystal structure (TmIGPS, PDB: 1I4N: SsIGPS, PDB: 2C3Z). No high-resolution structure has been reported for Eukaryotic IGPS.

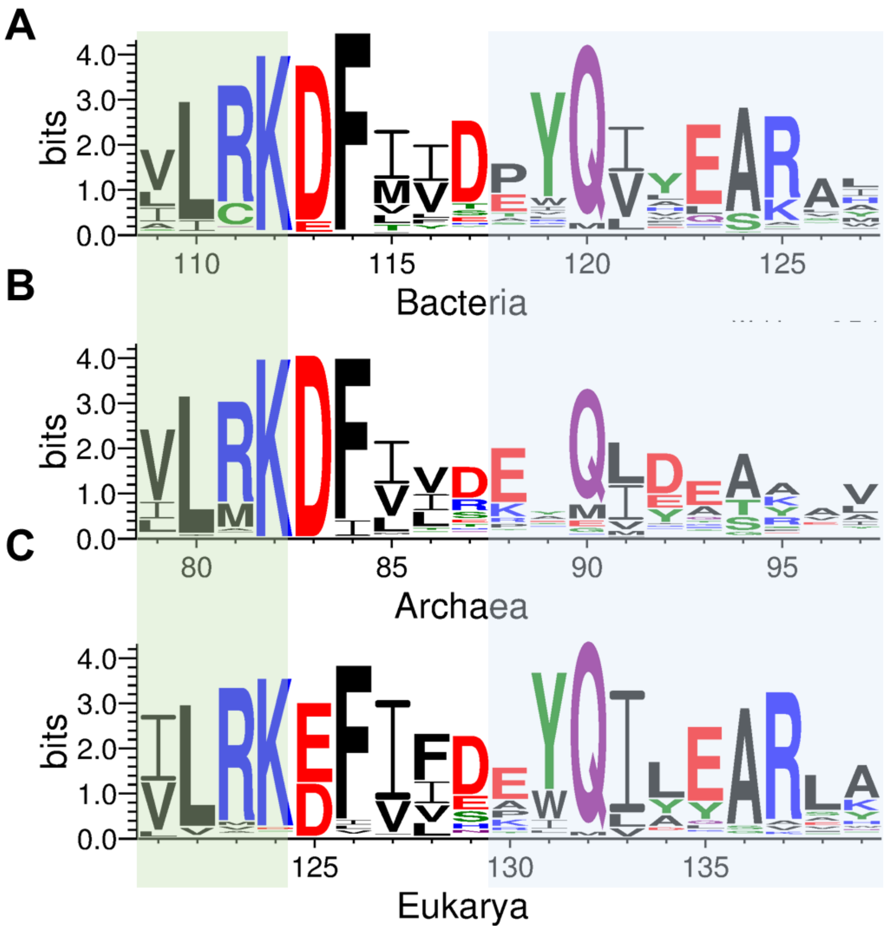

Fig. S14. Sequence logos for the aligned IGPS amino acid sequences in β3 (green) and α3 (blue) segments from (A) Bacteria, (B) Archaea and (C) Eukarya.

Dataset S1 (separate file). H-to-D exchange behavior and deuterium uptake of selected peptides from TmIGPS following proteolytic digestion of samples from equilibrium HDX-MS experiments at all experimental GdnHCl concentrations. The isotopic envelope is fitted and the secondary structure segments for peptides (Main Text, Figs. 4-5; SI Appendix, Fig. S7) are colored to represent the class of their H-to-D exchange behavior. Class I (red), Class II (green), Class III (blue) and Class IV (violet).

SI References

1. Gill SC & von Hippel PH (1989) Calculation of protein extinction coefficients from amino acid sequence data. *Analytical biochemistry* 182(2):319-326.
2. Nozaki Y (1972) The preparation of guanidine hydrochloride. *Methods in enzymology* 26:43-50.
3. Bilsel O, Zitzewitz JA, Bowers KE, & Matthews CR (1999) Folding mechanism of the alpha-subunit of tryptophan synthase, an alpha/beta barrel protein: global analysis highlights the interconversion of multiple native, intermediate, and unfolded forms through parallel channels. *Biochemistry* 38(3):1018-1029.
4. Jain R*, et al.* (2013) X-ray scattering experiments with high-flux X-ray source coupled rapid mixing microchannel device and their potential for high-flux neutron scattering investigations. *The European physical journal. E, Soft matter* 36(9):109.
5. Bai Y, Milne JS, Mayne L, & Englander SW (1993) Primary structure effects on peptide group hydrogen exchange. *Proteins* 17(1):75-86.
6. Kan ZY, Mayne L, Chetty PS, & Englander SW (2011) ExMS: data analysis for HX-MS experiments. *J Am Soc Mass Spectrom* 22(11):1906-1915.
7. Knochel T, Pappenberger A, Jansonius JN, & Kirschner K (2002) The crystal structure of indoleglycerol-phosphate synthase from Thermotoga maritima. Kinetic stabilization by salt bridges. *The Journal of biological chemistry* 277(10):8626-8634.
8. Schneider B*, et al.* (2005) Role of the N-terminal extension of the (betaalpha)8-barrel enzyme indole-3-glycerol phosphate synthase for its fold, stability, and catalytic activity. *Biochemistry* 44(50):16405-16412.
9. Kathuria SV, Chan YH, Nobrega RP, Ozen A, & Matthews CR (2016) Clusters of isoleucine, leucine, and valine side chains define cores of stability in high-energy states of globular proteins: Sequence determinants of structure and stability. *Protein science : a publication of the Protein Society* 25(3):662-675.
10. Radzicka A & Wolfenden R (1988) Comparing the polarities of the amino acids: side-chain distribution coefficients between the vapor phase, cyclohexane, 1-octanol, and neutral aqueous solution. *Biochemistry* 27(5):1664-1670.
11. Forsyth WR & Matthews CR (2002) Folding mechanism of indole-3-glycerol phosphate synthase from Sulfolobus solfataricus: a test of the conservation of folding mechanisms hypothesis in (beta(alpha))(8) barrels. *Journal of molecular biology* 320(5):1119-1133.
12. Yang X, Kathuria SV, Vadrevu R, & Matthews CR (2009) Betaalpha-hairpin clamps brace betaalphabeta modules and can make substantive contributions to the stability of TIM barrel proteins. *PloS one* 4(9):e7179.
13. El-Gebali S*, et al.* (2018) The Pfam protein families database in 2019. *Nucleic acids research* 47(D1):D427-D432.
