# Supplementary material for: A conserved folding nucleus sculpts the free energy landscape of bacterial and archaeal orthologs from a divergent TIM barrel family
Source: Proc Natl Acad Sci U S A. 2021 Apr 19;118(17):e2019571118. doi: 10.1073/pnas.2019571118 (PMC8092565; doi:10.1073/pnas.2019571118)
Supplement: Supplementary File [file pnas.2019571118.sd01.pdf]

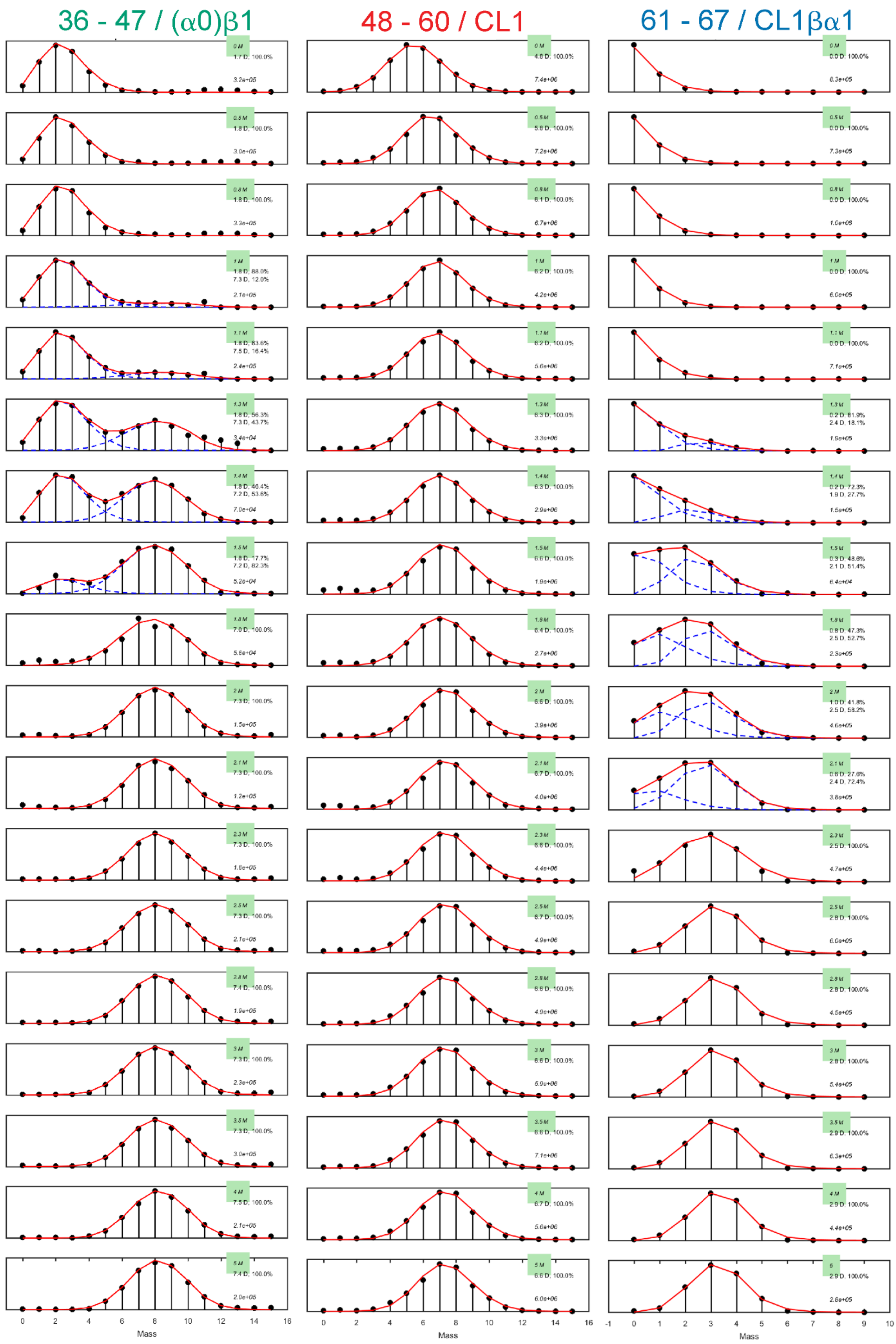

Supplementary Dataset S1

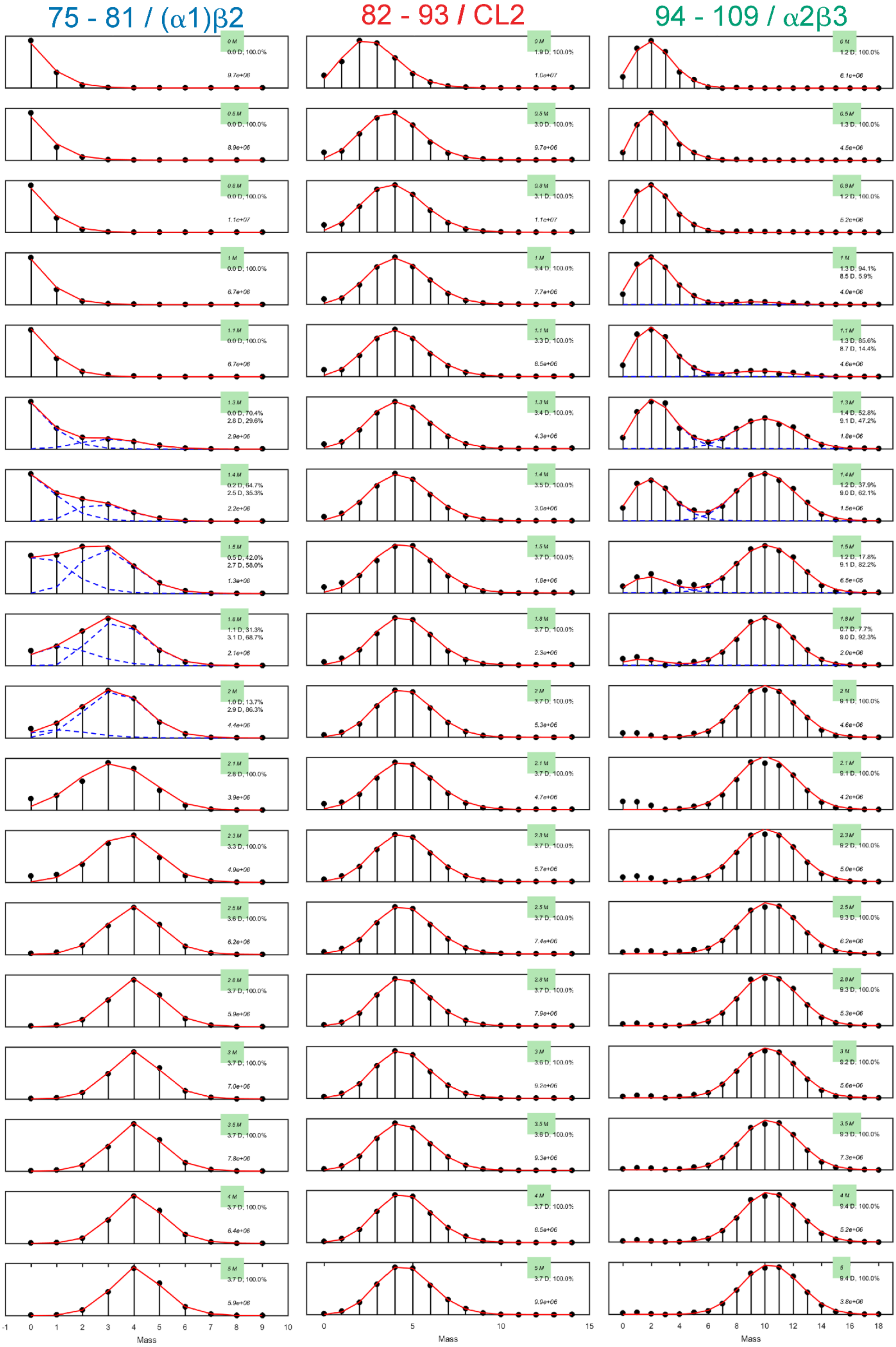

Supplementary Dataset S1

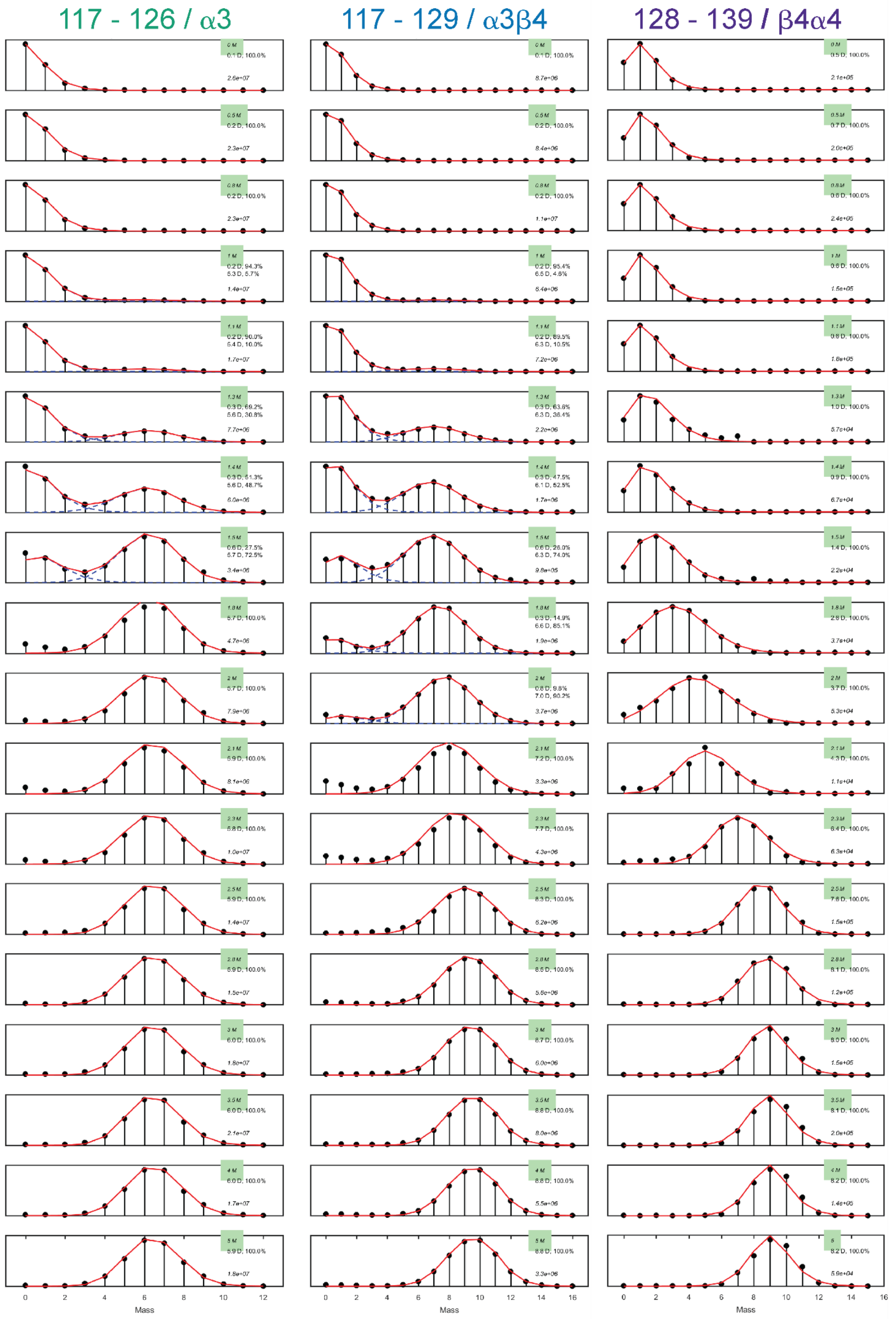

Supplementary Dataset S1

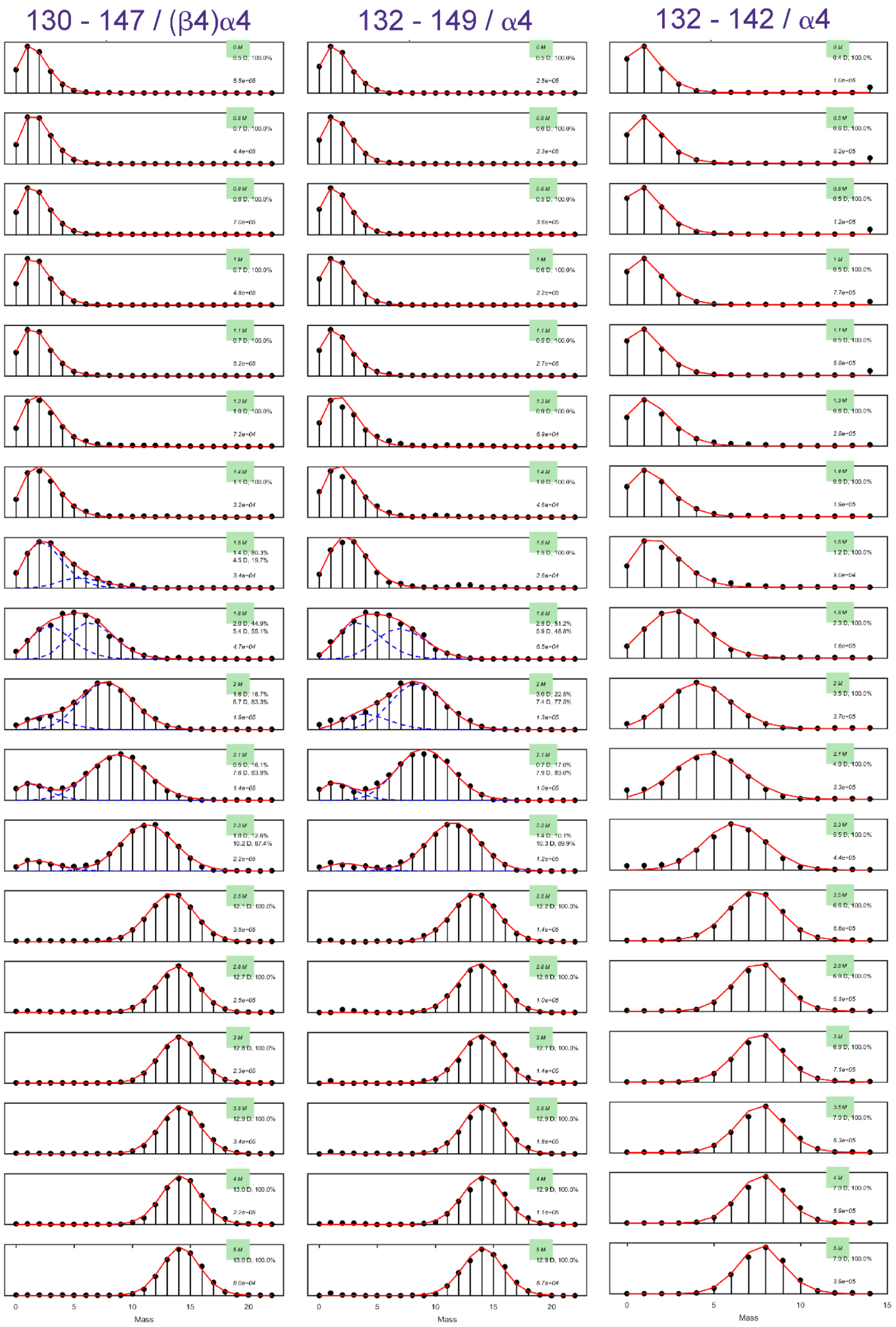

Supplementary Dataset S1

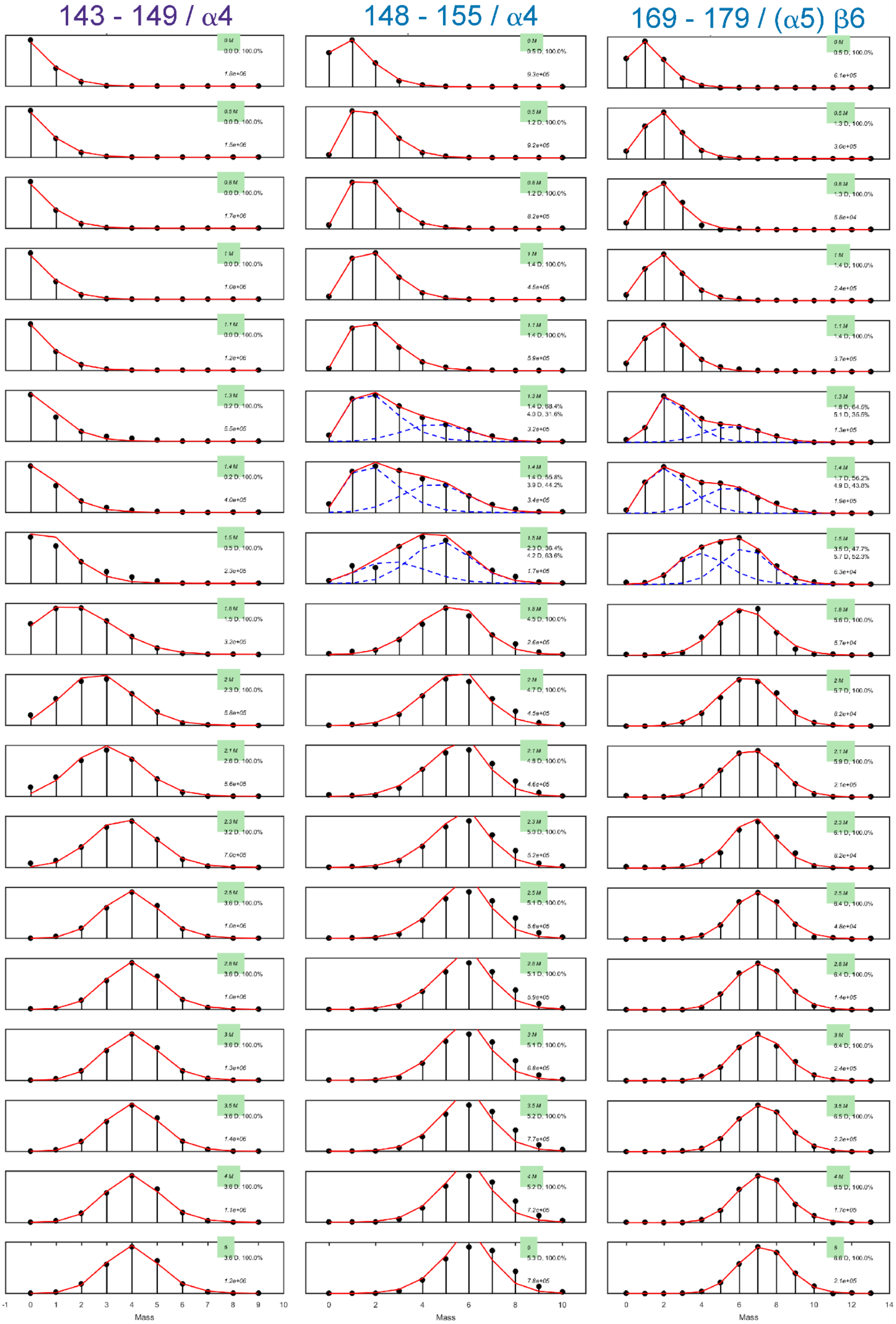

Supplementary Dataset S1

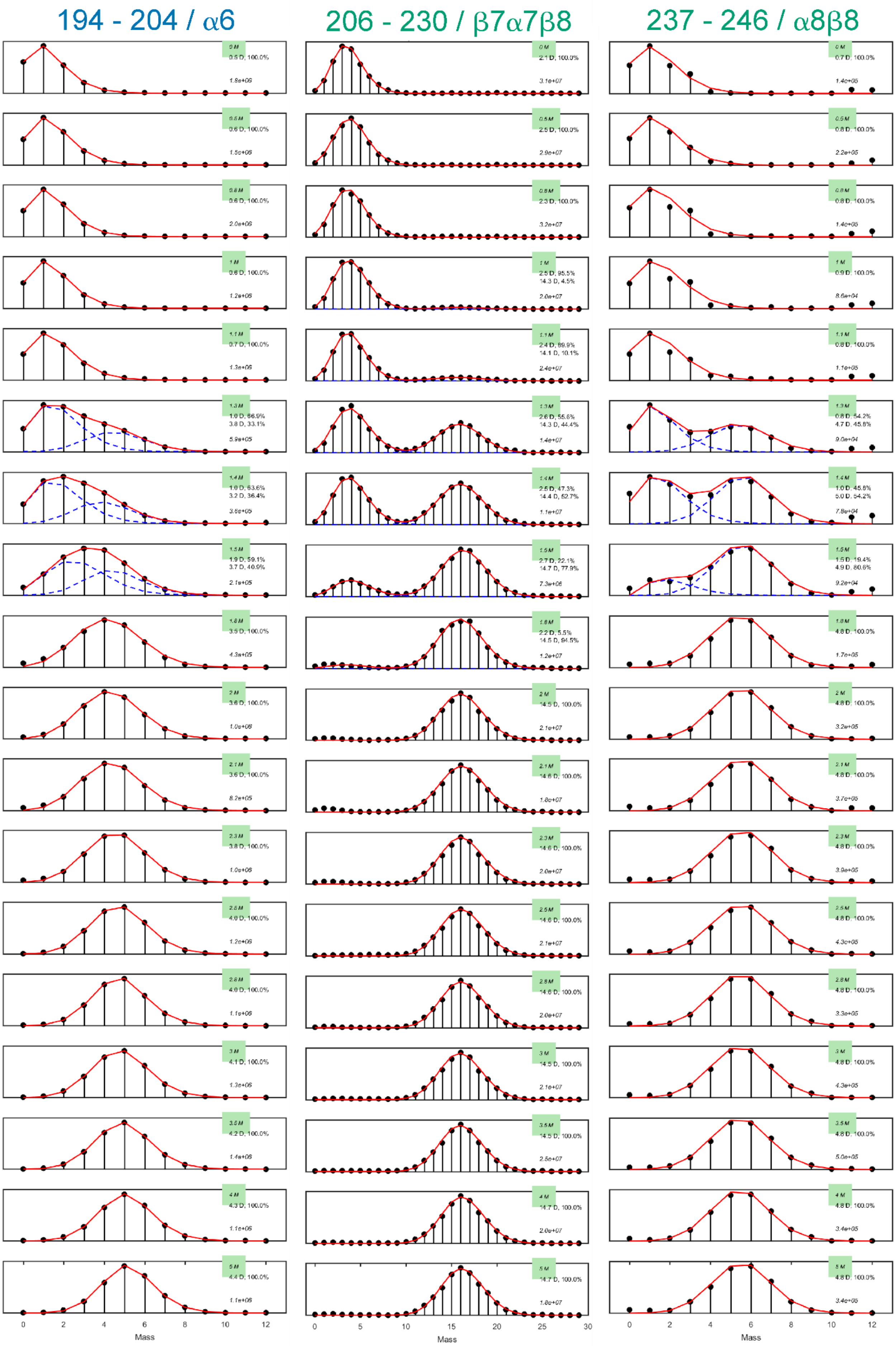

Supplementary Dataset S1
